# David Stafford-Clark (1916-1999): Seeing through a celebrity psychiatrist

**DOI:** 10.12688/wellcomeopenres.11411.1

**Published:** 2017-04-26

**Authors:** Gavin Miller

**Affiliations:** 1 School of Critical Studies/Medical Humanities Research Centre, University of Glasgow, Scotland, G12 8QQ, UK

**Keywords:** BBC, celebrity, David Stafford-Clark, psychiatry, public intellectual

## Abstract

This article uses the mass-media career of the British psychiatrist David Stafford-Clark (1916-1999) as a case study in the exercise of cultural authority by celebrity medical professionals in post-war Britain. Stafford-Clark rose to prominence in the mass media, particularly through his presenting work on medical and related topics for BBC TV and Radio, and was in the vanguard of psychiatrists and physicians who eroded professional edicts on anonymity. At the height of his career, he traded upon his celebrity status, and consequent cultural authority, to deliver mass media sermons on a variety of social, cultural, and political topics. Stafford-Clark tried to preserve his sense of personal and intellectual integrity by clinging to a belief that his authority in the public sphere was ultimately to be vindicated by his literary, intellectual, and spiritual significance. But as his credibility dwindled, he came to distrust the cultural intermediaries, such as broadcasters and publishers, who had supported him.

## Introduction

In the mid-1960s, a charismatic British psychiatrist rose to prominence in the mass media. Impelled by a desire for literary and cultural significance, he exploited his psychiatric authority and celebrity status to comment on a wide range of social, cultural, and political matters. As the years passed, and his celebrity dwindled, he resorted to popular journalism and bad poetry to air his unfashionable views. His name was R.D. Laing (1927–1989), and he was the *enfant terrible* of the countercultural anti-psychiatric movement.

In the late-1950s, a charismatic British psychiatrist rose to prominence in the mass media. Impelled by a desire for literary and cultural significance, he exploited his psychiatric authority and celebrity status to comment on a wide range of social, cultural, and political matters. As the years passed, and his celebrity dwindled, he resorted to popular journalism and bad poetry to air his unfashionable views. His name was David Stafford-Clark (1916–1999), and he was the Establishment voice of mainstream psychiatry.

Laing’s determined pursuit of celebrity, and his delight in it, fulfilled a longstanding ambition established in his teenage years ( Beveridge, 2011: 38–44, 315–316). This ambition came to fruition in the 1960s, with Laing’s first TV appearances in 1963 ( Laing, 2006: 81–82), leading to further broadcast and media work in the UK ( Laing, 2006: 84). Such domestic exposure facilitated what was, by 1967, his international celebrity standing: Laing’s ‘popularity seemed limitless, fuelled as it was by his active work in his field, his powerful charisma, intellectual reputation, and uncanny rapport with the rebellious anger of the 1960s’ ( Laing, 2006: 134). Daniel Burston identifies the ‘corrosive impact of fame on R.D. Laing’ ( Burston, 1996: 75) at a personal level, and these effects were also apparent intellectually. As Adrian Laing indicates, Laing’s pursuit of celebrity distracted him from rigorous intellectual activity: ‘Ronnie’s “stuff” was brilliant and exciting the first time round; listening to his views more than once made them seem platitudinous, bordering on the self-indulgent’ ( Laing, 2006: 109). A similar pattern has been described previously, whereby Laing’s marketability for the UK publisher Penguin encouraged them to turn a blind eye to the potential shortcomings of his written output ( Miller, 2015: 86–91). By 1977, Laing’s diminishing psychiatric significance meant that he ‘wished to be regarded more as a poet’ ( Laing, 2006: 191), but he was unable to fulfil this literary ambition, producing poorly reviewed bottom-drawer efforts such as *Do You Love Me?: An Entertainment in Conversation and Verse* ( Laing, 1977).

There were undoubtedly a number of post-war British psychiatrists who, like Laing and Stafford-Clark, exercised wider cultural authority in the public sphere. G.M. Carstairs (1916–1991), for instance, delivered in 1962 the British Broadcasting Corporation’s (BBC) annual Reith Lectures, initially broadcast on radio, and later published as *This Island Now* ( Carstairs, 1963). Carstairs followed an illustrious line of social and cultural commentators – including Peter Medawar, Arnold Toynbee, and Bertrand Russell – and offered ‘nothing less than a review of the State of the Nation, in the light of changes which have come about in the community and in private life since the beginning of the century’ ( Carstairs, 1963: 7). He employed a social anthropological gaze upon an increasingly post-colonial Britain, so that ‘the skills used in studying “national character” can be directed towards throwing light on areas of malaise in our own society’ ( Carstairs, 1963: 22). He touched upon a number of contentious areas – including the arms race ( Carstairs, 1963: 96–98) – and found himself embroiled in controversies over pre-marital sexuality: ‘The implication that before long premarital sexual intercourse, with safeguards against conception, may become part of the experience of every maturing boy or girl raised a storm of protest, and a counter-demonstration from those who welcome this eventuality’ ( Carstairs, 1963: 9). Nor was such commentary solely the province of a social psychiatrist like Carstairs. Further intervention in wider culture came from William Sargant – again, an occasional broadcaster – who was ‘one of the foremost exponents of the physical methods of treatment that emerged in 20th century psychiatry’ ( Beard, 2009: 28). In successful mass-market books, such as *Battle for the Mind* ( Sargant, 1957) and *The Mind Possessed* ( Sargant, 1973), Sargant surveyed folk and expert technologies for ideological conversion, drawing a continuity between ‘the doctor and his nervous patient’, ‘the religious leader who sets out to gain and hold new converts’, and ‘whole groups of nations, who wish not only to confirm certain political beliefs within their boundaries but to proselytize the outside world’ ( Sargant, 1957: 19).

Carstairs and Sargant, while successful authors in the book market, and occasional broadcasters, did not rival Laing’s degree of celebrity. Stafford-Clark, though, by virtue of his temporary status as ‘the BBC’s psychiatrist’ (see below), rivalled at a national, British level, the later level of exposure enjoyed by Laing. From 1957–1962, Stafford-Clark presented almost 60 episodes of the BBC TV programme *Lifeline*, at a time when audience figures for the UK’s two TV channels (BBC and ITV) could easily run into millions. Laing’s trajectory as a celebrity – his widely discussed rise and fall in public consciousness, his diminishing intellectual credibility, and his failure as a creative (rather than discursive) writer – therefore invites questions that can be answered by a detailed analysis of his precursor, Stafford-Clark, who is, unlike Laing, generally forgotten, and academically neglected (excluding Miller, 2015: 79–83; Wells, 1995: 64, 190–191; Long, 2014: 37–38; Jones, 2006: passim). Laing’s first British TV appearances in 1963–1964 ( Laing, 2006: 81–84) occurred just as Stafford-Clark was being sidelined by the BBC, the British state-funded broadcaster that had propelled him onto the national stage and into the homes of viewers and listeners. Laing ended up filling the mass-media celebrity psychiatrist role that Stafford-Clark was being forced to vacate, and which the latter had been instrumental in creating, including through the erosion of professional edicts on media anonymity. Stafford-Clark’s career illuminates the cultural economy and the biographical factors that attend the creation of celebrity psychiatrists in the mass media. Rich archival material, in the form of personal papers and social documents (see below), allows detailed study of Stafford-Clark as a fame-hungry psychiatrist negotiating – sometimes unwisely – with the demands of broadcast and publishing markets, and with the economy of celebrity.

Stafford-Clark was a talented lecturer and broadcaster, albeit something of a *prima donna*, who rose to prominence in the post-war British mass media, particularly through his presenting work for BBC TV and Radio, where he exercised his facility for the more convivial, conversational persona demanded by the new broadcasting media. At the height of his career, he traded upon his celebrity status to deliver mass-media sermons on a variety of social, cultural, and political topics, frequently exceeding the limits of his psychiatric expertise. Like his successor, Laing, Stafford-Clark was increasingly over-exposed, and trading on a diminishing intellectual capital. Laing hoped for a literary success that would renew his cultural standing. In similar fashion, Stafford-Clark tried to preserve his sense of personal and intellectual integrity by clinging to a belief that his authority in the public sphere was ultimately to be vindicated by his literary, intellectual, and spiritual significance (rather than being in large part ascribed by the BBC’s exploitation of his talent for radio and television presenting). As Stafford-Clark’s celebrity dwindled, though, he came to distrust the very cultural intermediaries that had supported him: broadcasters, publishers, editors, newspapers, and so forth, putatively distorted, disregarded, and even censored, his views.

## Stafford-Clark’s life and medical career

David Stafford-Clark was born 17 March 1916 to a middle-class family (his father was a lawyer ( Stafford-Clark, 1957: 65)), and privately educated on a scholarship as a boarding pupil at Felsted School in the South of England (PP/DSC/A/1 c.v., May 1982 ^1^). He took his medical degree at Guy’s Hospital Medical School in the University of London, graduating MBBS in May 1939 (PP/DSC/A/1 c.v., May 1982). On the outbreak of war, Stafford-Clark volunteered for service, and became a Medical Officer in the RAF. Research on aircrew in Bomber Command led to his post-war article ‘Morale and Flying Experience’ ( Stafford-Clark, 1949), as well as a lengthy, although unexamined, research thesis ( Stafford-Clark, 1987: 219). Stafford-Clark’s younger brother, who also enlisted, died in a flying accident in 1941 while serving in the Fleet Air Arm ( Stafford-Clark, 1987: 218). This profound bereavement spiritually transformed Stafford-Clark, who rediscovered his lapsed Christian faith ( Stafford-Clark & Comfort, 1967: 123). In 1942, after three unsuccessful proposals, Stafford-Clark was finally engaged and married to Dorothy Stewart ( Stafford-Clark, 2014: 38), a war widow with a young son, Max (later to become a highly-regarded theatre director).

 After the war, Stafford-Clark completed his training in general medicine at Guy’s, gaining his MRCP in 1946, and his MD (without thesis) in 1947 ( Stafford-Clark, 1987: 219). He then used a three-year Nuffield Fellowship to specialize in psychiatry at The Maudsley Hospital, spending a final year in the USA. Upon returning in 1950, he worked briefly at The Maudsley, before moving to Guy’s to become Assistant Physician in the Department of Psychological Medicine, and Deputy Director of the York Clinic (PP/DSC/A/1 c.v., May 1982). In 1955, having dedicated himself primarily to undergraduate teaching alongside his clinical work, Stafford-Clark was promoted to Physician in Charge in Psychological Medicine, and Director of the York Clinic (PP/DSC/A/1 c.v., May 1982). He held these positions, alongside other clinical and academic appointments, until a severe allergy acquired during wartime trials with gas warfare led to a near-fatal event in 1971 ( Stafford-Clark, 1987: 223). He was forced to gradually relinquish his professional commitments, and finally took early retirement in 1974 at age 58 ( Stafford-Clark, 1987: 223). He then moved to healthier climes in Northern Cyprus, until his health recovered sufficiently for a return to the UK in 1982 ( Stafford-Clark, 1987: 223), where he lived until his death in September 1999 ( anon., 1999).

 Stafford-Clark’s intra-disciplinary contribution to psychiatry came largely through his clinical, educational and organizational leadership, rather than through research. Ironically, one of his most significant research contributions occurred before his formal psychiatric training. As an RAF Medical Officer, he was prominent amongst those working within Bomber Command to develop a more humane and psychologically informed response to aircrew stress ( Wells, 1995: 64, 190–191). Stafford-Clark distrusted the quasi-medical categorisation ‘Lack of Morale Fibre’ (LMF; Jones, 2006: 439–441), a stigmatising, psychiatrically ill-informed, and inflexible designation introduced early in the war, and which was, in effect, an attribution of cowardice ( Jones, 2006: 441–445). Stafford-Clark’s research on aircrew morale, which was published after the war as a lengthy article in *Journal of Mental Science* ( Stafford-Clark, 1949), offers a more nuanced fourfold typology of psychogenic responses to stress in aircrew. He carefully distinguishes symptoms arising naturally from the progression through an operational tour, as well as those arising because of exceptional stress ( Stafford-Clark, 1949: 23–27). This leaves over a small fourth category of cases that might otherwise have been designated as LMF, but which are described by Stafford-Clark as those whose ‘morale is intrinsically poor’ – such men are ‘essentially unwilling to fly’ and should be promptly removed from operational service ( Stafford-Clark, 1949: 29).

Nonetheless, while he published various post-war research papers (e.g. Clarkson & Stafford-Clark, 1960; Stafford-Clark, 1949; Schwab *et al*., 1951), with a particular interest in psychopathy (e.g. Stafford-Clark *et al*., 1951; Gibbens *et al*., 1955), Stafford-Clark’s professional focus as a psychiatrist was the Department of Psychological Medicine and the in-patient York Clinic at Guy’s. The latter, which offered a convivial ambience to its patients, had been established in 1944 by R.D. Gillespie, and drew upon his wartime experience with RAF aircrew who had suffered breakdown ( Jones, 2004: 503): ‘Although not specifically a therapeutic community, the liberal regime and range of treatments offered marked a change from the asylum culture and represented a conscious attempt to raise the status of psychiatry and its allied disciplines’ ( Jones, 2004: 503). Stafford-Clark describes Psychological Medicine and the York Clinic at Guy’s as pioneering ‘the integration of psychiatric services within a general teaching hospital’ ( Stafford-Clark, 1961a: 4). He lauds the ease with which other Guy’s units can refer patients ( Stafford-Clark, 1961a: 10), and the opportunity to allow all trainees ‘a foundation for understanding human personality, and its repercussion upon physiological function’ ( Stafford-Clark, 1961a: 13). Stafford-Clark’s philosophy was to promote ‘eclectic clinical psychiatry’ ( Stafford-Clark, 1987: 220), including electroconvulsive therapy (ECT), electro-narcosis, and ‘the judicious use of psychopharmacology’, as well as ‘psychotherapy, both supportive and intensive’ ( Stafford-Clark, 1987: 220). He was unpersuaded by anti-psychiatry – as shown in a dismissive review of Thomas Szasz ( Stafford-Clark, 1964b) – but clearly valued psychotherapy, whether as adjunct ( Stafford-Clark & Willis, 1959: 538), or as depth psychoanalysis for a minority of cases ( Stafford-Clark, 1965b: 215). He was thus positioned therapeutically in a middle ground between the heroic somatic interventions favoured by the likes of William Sargant ( Beard, 2009: 26–28), and the radical distrust of ECT, surgery, and psychopharmacology propounded by Laing and the anti-psychiatrists. Stafford-Clark thus strongly defended insulin coma therapy for schizophrenia ( Stafford-Clark, 1955: 8), and continued to use insulin, even after it was discredited, ‘as an adjuvant in the treatment of acute and chronic anxiety, and anorexia nervosa in selected cases’ ( Stafford-Clark, 1987: 220).

## Stafford-Clark’s mass-media career

Stafford-Clark’s mass-media career, the focus of this article, flourished in the 1950s as the medical profession began to find a more collaborative relationship with broadcasters and mass-market publishers (see below). His career as a mass-market author began in 1952 with his Penguin popular introduction, *Psychiatry To-day* ( Stafford-Clark, 1952), which eventually sold over 100,000 copies, and remained in print until the early 1970s ( Miller, 2015: 81). Stafford-Clark’s broadcasting career began soon thereafter, in 1953, with radio work for the BBC. His BBC TV career started in 1955, before rapidly flourishing via the series *Lifeline* (1957–1962), through which, cloaked in threadbare anonymity, he became *de facto* ‘the BBC’s psychiatrist’. At the height of his fame in the late 1950s and early-to-mid 1960s, Stafford-Clark was a national celebrity – a familiar face to millions, and a recognized name to many, despite the supposed veil of professional anonymity. Two further series followed, *Brain and Behaviour* (1964), and *Mind and Motive* (1967), alongside miscellaneous TV work. Stafford-Clark also wrote for periodicals and newspapers, including *The Listener* ( Stafford-Clark, 1959b), *Nova* ( Stafford-Clark, 1966a) and *The Sunday Times* ( Stafford-Clark, 1967b), and was a frequent invited speaker. His career as an author continued in the textbook *Psychiatry for Students* ( Stafford-Clark, 1964c), through various editions, a widely-translated popular introduction called *What Freud Really Said* ( Stafford-Clark, 1965b), which remained in print until the 1990s ( Stafford-Clark, 1997), and a post-retirement novel, *Soldier Without a Rifle* ( Stafford-Clark, 1979).

In psychiatric contexts, Stafford-Clark described his mass-media work as resulting from an enthusiasm for ‘public education in psychiatry and the immense opportunities for it in writing, radio and ultimately television’ ( Stafford-Clark, 1987: 220). The need for public education was clearly revealed in post-war studies of the stigma attached to mental illness, such as Enid Mills’ research in the late 1950s on hospitalized mental patients in East London. Mills noted how ‘[m]any of the patients recognized that there was something the matter with them, but denied its nature’ ( Mills, 1962: 43) – a denial with which close relatives frequently colluded because they were ‘chary of openly facing something which they fear as a stigma on their family’ ( Mills, 1962: 48). The need for a psychiatrist-led campaign of education against stigmatization seemed pressing in the post-war period (though admittedly later research has challenged the assumption that promoting the medical model of mental illness necessarily reduces stigma (e.g. Read & Law, 1999)). As Vicky Long notes, Stafford-Clark’s activities were informed by a fundamental model of public deficit (c.f. Hilgartner, 1990): ‘If mental illness was stigmatised then, in Stafford-Clark’s eyes, the fault lay with an ignorant public’ ( Long, 2014: 38). This was part of a general pattern whereby ‘psychiatrists focused on raising the status of their profession and tended to treat the public as an apathetic and homogeneous mass. Ignorant, resistant to the careful efforts of psychiatrists to educate them and yet strangely prone to sensationalist reporting, the public as envisaged by psychiatrists were not capable of participating in a debate about the care of the mentally ill’ ( Long, 2014: 50).

Further examination of Stafford-Clark’s oeuvre in various media confirms his cautious view of the potential for public education, and his tendency to dwell on the stigmatization of psychiatrists, rather than their patients. In *Psychiatry To-day*, Stafford-Clark explains that ‘[w]e can liken the whole development of psychiatry, and its impact upon ordinary men and women, to the discovery and exploration of a volcano upon a desert island. For a very long time the islanders have lived with the volcano in their midst’ ( Stafford-Clark, 1952: 296). The primitive islanders (the lay public) ‘transfer a good deal of their mingled feelings of hate and fear and worship from the volcano itself to their explorers’ ( Stafford-Clark, 1952: 297). Stafford-Clark was thus convinced that public education in psychiatry had to overcome a commercial demand contaminated by the displaced emotions that surrounded mental illness. In a 1958 article for the cinema magazine *Films and Filming*, Stafford-Clark, thinly veiled as ‘a famous consultant psychiatrist, working in a London clinic’, remarks that psychiatry ‘tends to arouse in the public a curious and interesting mixture of dread, derision – and demand’ for reasons dealt with ‘in a book I once wrote’ ( Stafford-Clark, 1958: 8). Commodified cultural productions such as ‘[t]he film industry’ and ‘most of the newspapers’ are bound ‘to follow established conventional patterns, seemingly imposed by the expectations of its audience’, and thus cater to the audience’s everyday psychopathologies: ‘screen psychiatry is customarily presented as material for awe, ridicule – or magical fulfilment’ ( Stafford-Clark, 1958: 8).

 Stafford-Clark’s analysis of public education in psychiatry was therefore pessimistic: the public were predisposed to hate, fear, and even worship psychiatry; moreover, popular culture catered to their mass neurotic ignorance for reasons of commercial profitability. The psychiatrist’s later personal experience of commercial film-making may have been salutary, particularly his collaboration with the director John Huston on the latter’s 1962 biopic, *Freud: The Secret Passion*. The film’s screenwriter, Jean-Paul Sartre, had delivered a script that ‘could have run to sixteen hours of film’ ( Meyers, 2011: 184), even though he had chosen ‘to telescope some dozen or so of Freud’s case studies into a single, decisive one’ ( Shortland, 1987: 435). Huston, with his script doctor Wolfgang Reinhardt, laboured to condense Sartre’s screenplay into a length suitable for mass-market cinematic release. Stafford-Clark, at that time nationally famous from *Lifeline*, was called in ‘to give expert counsel’ ( Shortland, 1987: 435). (Indeed, according to Meyers, Stafford-Clark was required again during filming to treat its troubled star, Montgomery Clift ( Meyers, 2011: 191).) Stafford-Clark’s views on *Freud* are unclear, but he would certainly have noticed Huston ‘adapting the storyline to that other popular genre, the detective story’, so that ‘the psychoanalyst in taking the role of the detective consigns to the patient the role of the witness who will not talk’ ( Shortland, 1987: 439). Moreover, even the post-Sartre version was edited further after test screening of the director’s cut: ‘previews to invited audiences led to major cutting, as did reaction from studio executives who considered that some scenes were morally offensive and would provoke organized reaction’ ( Shortland, 1987: 435).

*Freud* could well have confirmed Stafford-Clark’s impatience with commercial cinema, and may have encouraged his collaboration on three specialist documentary productions with the film maker Eric Marquis (1928-): *And Then There Was One* (1962), *Time Out of Mind* (1966), and *The Savage Voyage* (1971) (PP/DSC/B/4/1). Documentary film might have seemed an ideal way to educate the public about the reality of mental illness and the nature of the psychiatric profession. However, these productions, in which Stafford-Clark took the role of advisor and commentator, were industrial films, sponsored by pharmaceutical manufacturers and intended only for medical professionals. Roche Products, the pharmaceutical sponsor of *Time out of Mind*, describe the film as projecting ‘the academic and clinical knowledge of a consultant psychiatrist through the creative skill and talent of a writer and film director. This marriage of two disciplines enables the audience to share in a disturbing, but clinically illuminating way, the psychiatric patient’s whole experience of breakdown, treatment and recovery’ (cited in Vick, 2010: 377). In this short film, ‘three emotionally disturbed patients’ case histories are dramatically reconstructed’ ( Vick, 2010: 377–378) using experimental techniques to convey the first-person experience of mental illness. The film was a critical success, ‘winning […] the Industrial Film Correspondent Director’s Award, a Silver Award from the British Medical Association and a much sought-after BISFA Gold Award’ ( Vick, 2010: 377), but was only available ‘[o]n restricted loan to those working in the field of psychiatry’ ( Vick, 2010: 378), so its wider impact on ‘public education’ was almost negligible.

 Stafford Clark’s motivations for his mass-media work were thus undoubtedly less altruistic, and more complex, than merely ambivalent service to the public education in psychiatry, whether conceived as the destigmatisation of mental illness, or the removal of myths surrounding the psychiatric profession itself. Max Stafford-Clark states his father had ‘a strong element of the showman’, apparent in a remark that ‘he had become a doctor so he could be the person who made his way through the crowd in the event of an accident’ ( Stafford-Clark, 2014: 37). He recalls ‘seeing him on a ward round looking like a medieval duke surrounded by a court of nurses and junior doctors’ ( Stafford-Clark, 2014: 37–38) – indeed, the theatre ‘was a career he admired and would have liked in some form for himself’ ( Stafford-Clark, 2014: 38). Stafford-Clark’s need to be in the public eye was certainly gratified by a mass-media career that made him into a national celebrity. A brief vignette illustrates his prominence: in a 1965 magazine article, Stafford-Clark records indignantly the storm of press coverage (including being door-stepped by reporters) evoked by a lecture to the Scottish Marriage Guidance Council where he had cited bawdy songs in order to ‘exemplify both the logic and the grounds of my conviction about the implications of pornography’ ( Stafford-Clark, 1965a: 20). Because of national press coverage following a local councillor’s complaint, there arose mistaken perceptions of the lecture: ‘many people’s first impression was that I had either recited the poem “Eskimo Nell” (longest extant version 58 verses), or sung the song about the airman with the sexually insatiable wife’ ( Stafford-Clark, 1965a: 18). *The Guardian* dutifully reported Stafford-Clark’s defence that ‘he had recited parts of “Eskimo Nell” not as a joke but in an attempt to illustrate the kind of language so many people use about sex. Sex was a “beautiful thing”, which should be spoken about with “reverence”’ ( anon., 1965). With typical ambivalence, Stafford-Clark laments the ‘degree of absolute distortion which had found its way into the news story’ ( Stafford-Clark, 1965a: 21). This reference to ‘distortion’ by the mass media articulates the well-known scientific folk mythology critiqued by Stephen Hilgartner, in which ‘any differences between genuine and popularized science must be caused by “distortion” or “degradation” of the original truths’ by scientific outsiders, such as policy-makers, the public, and the mass media ( Hilgartner, 1990: 519). Yet, as a celebrity psychiatrist with a taste for showmanship, Stafford-Clark clearly invited ‘distortion’ with his flamboyant displays, such as reciting the story of how ‘When Dead-Eye Dick and Mexican Pete set out in search for fun, / It’s Dead-Eye Dick who wields the prick and Mexican Pete the gun’ ( Stafford-Clark, 1965a: 20).

A classification proposed by Chris Rojek illuminates Stafford-Clark’s ambivalent relationship to his own celebrity. Rojek distinguishes between *ascribed*, *achieved*, and *attributed* celebrity ( Rojek, 2001: 17–20). The former, based on lineage and bloodline, is irrelevant to Stafford-Clark. However, the distinction between achieved celebrity and attributed celebrity is illuminating. The former ‘derives from the perceived accomplishments of the individual in open competition’ ( Rojek, 2001: 18); the latter arises from ‘the concentrated representation of an individual as noteworthy or exceptional by cultural intermediaries’ ( Rojek, 2001: 18). Stafford-Clark was unwilling to accept that his celebrity was to a large extent attributed by the mass-media machinery that employed him temporarily because of his talent for broadcasting, rather than achieved by lasting success in some other realm of human accomplishment.

In his desire to be well-known for something other than his well-knowness, Stafford-Clark maintained a lifelong conviction that he was ultimately to be recognized as ‘an unrecognized literary author of substantial merit’ ( Miller, 2015: 81). Stafford-Clark’s belief in his prodigious talent seems to have crystallized when he received in 1933 ‘the Gate Prize for poetry’, given ‘annually to “the best poem published in a school magazine”’ ( Stafford-Clark, 1987: 218). However, the response to Stafford-Clark’s volumes of wartime poetry, *Autumn Shadow* (1941) and *Sound in the Sky* (1944) was lukewarm: Stafford-Clark’s poetry was ‘proudly committed to patriotic, political and spiritual statements in a time of national crisis’ ( Miller, 2015: 82), but lacked poetic technique. When in 1944 Stafford-Clark sends his work to Siegfried Sassoon (1886–1967), the distinguished poet and novelist of World War One, the latter explains ‘many of your passages are blank verse, with lines occurring which don’t scan at all unless one ignores your line endings – and then there are loose bits left over. Don’t forget that form is essential to all art’ (PP/DSC/E/1 letter: Siegfried Sassoon, DSC, 12 December 1944). Stafford-Clark responded to such constructive literary criticism with a ferocious assertion of his literary excellence. Miller describes, for instance, Stafford-Clark’s tense relationship with Penguin editorial staff, including his aggrieved response to rejection in a nationwide poetry competition run by Penguin in 1951 ( Miller, 2015: 81). Stafford-Clark subsequently wrote directly to Allen Lane, Penguin’s founder and director, to propose a single-author volume of his poetry. An answer came via A.S.B. Glover, Stafford-Clark’s editor: ‘no publisher who […] has to depend on sales of 30,000 or 40,000 copies, could favourably consider for separate publication the work of any poet who had not a contemporary established reputation which put him among the first two or three of our recognised poets’ (PP/DSC/E/1 letter: ASB Glover, DSC, 3 December 1951). The request reveals Stafford-Clark’s unreasonably high self-estimation as the presumed poetic equal of Eliot or Auden (and also shows his patrician appeal to Lane over Glover’s head).

 As further opportunities arose in broadcasting, so Stafford-Clark continued to stubbornly promote his own poetry, hoping to be transmuted from the base metal of broadcasting. Less than a year after beginning on BBC Radio, his verse was scrutinized by P.H. Newby, the producer in charge of poetry broadcasting (RCONT1 letter: Richard Tatlock, DSC, 17 September 1954 ^2^). Newby criticises the ‘moralising’ tone of the poems, and the conventionality of the diction (RCONT1 ms annotation, memo: Richard Tatlock, PH Newby, 9 September 1954). Stafford-Clark reacts with typical ire, characterising this refusal as ill-mannered and indicative of the poor quality of contemporary poetry; with typical poetic vanity, he argues that Milton was equally moralistic (RCONT1 letter: DSC, Rev. Richard Tatlock, 20 September 1954). After at least one more unsuccessful pitch (RCONT1 Letter: Jocelyn Ferguson, DSC, 23 February 1962), Stafford-Clark was eventually granted in 1964 a 5-minute recording (RCONT1 letter: DSC, Rev. Elsie Chamberlain, 17 March 1964) broadcast on a magazine programme aimed at the elderly, and a final triumph arrived in a 30-minute valedictory autobiographical broadcast in 1973, in which a selection of his poems were recited (RCONT15 letter: Peter de Rosa, DSC, 19 February 1973).

Print, as well as broadcast work, provided a Trojan horse for Stafford-Clark’s poetic onslaught. As Miller notes, Stafford-Clark’s 1979 novel, *Soldier without a Rifle*, begins with an epigraph juxtaposing his poetry with that of the First World War poet, Wilfred Owen ( Miller, 2015: 81). The cited poem is even smuggled into the fictional world of the novel, where it appears (in full) as a work written by one of the central characters ( Stafford-Clark, 1979: 87–89). Stafford-Clark also exploited his 1970 Nelson Lectures in the University of Lancaster. The companion volume, *Five Questions in Search of an Answer* ( Stafford-Clark, 1970), might equally have been entitled *David Stafford-Clark: Selected Poems and Writings*, since it reproduces, in whole or in part, a number of his poems. Stafford-Clark alludes, for instance, to an unpublished collection, composed *c*.1949, before generously citing in full (over 11 pages) the Eliotean titular poem. In ‘The Way to the Battle’, a military parachute jump provides the central metaphor for ‘[a]n act of faith beyond evasion/A step from which – once made –/No turning back is possible’ ( Stafford-Clark, 1970: 56).

There is no case for a literary re-evaluation of Stafford-Clark, who remains a very minor footnote to the British canon. However, the psychiatrist’s *idée fixe* illuminates his negotiation with the celebrity status that he both desired and repudiated. Stafford-Clark was propelled into a position of ‘cultural authority’ ( Collini, 2006: 47–48) by Penguin’s mass publishing, and then by his national exposure on BBC TV and Radio. By his access to such media, he was empowered, and indeed incentivized, to speak to ‘general concerns’ ( Collini, 2006: 56) of the wider public audience via his views on political, ethical, and spiritual issues, amongst others. This career trajectory contrasts somewhat with the public intellectual role outlined by Collini, where the intellectual’s expression of views on matters of general concern depends upon a prior ‘“qualifying” activity’, ‘a level of achievement in an activity which is esteemed for the non-instrumental, creative, analytical, or scholarly capacities it involves’ ( Collini, 2006: 52). Crucially, this ‘achievement or proficiency in a sphere of activity loosely recognized as “intellectual”’ occurs ‘ *independently* of the activity of speaking out on public issues’ ( Collini, 2006: 47, 48). To use just a few of Collini’s case studies: T.S. Eliot was a distinguished poet before he was an intellectual; A.J.P. Taylor, an accomplished academic historian; Bertrand Russell, a leading philosopher; and so forth. Stafford-Clark’s claim to a qualifying activity was, however, less secure. He had been helped into the paperback publishing market at an early stage of his career by the patronage of the distinguished psychiatrist Aubrey Lewis, who facilitated an introduction to Penguin in 1949 ( Miller, 2015: 80). Stafford-Clark’s psychiatric gifts were more for communication (and organizational leadership) than for innovation: *Psychiatry To-day* succeeded because Stafford-Clark’s talent for exposition, and enthusiasm for public prominence, found a ready audience in ‘the emerging postwar market of British psychologized subjects’ ( Miller, 2015: 81).

Stafford-Clark was partially aware that the cart of celebrity had come before the horse of achievement: having had greatness thrust upon him, he was in search of some independent ‘qualifying activity’ that would legitimate his cultural authority as his own achievement. At times, he could be disarmingly frank about his own limitations. In a 1966 magazine article on sex manuals, he admits that ‘as the author of a paperback which has itself sold steadily and been reprinted a number of times, I cannot range myself anywhere but with those whose works I have been considering’ ( Stafford-Clark, 1966b: 27). Stafford-Clark compares the ‘earnest scribes’ behind these books, and by extension himself, to ‘low-handicap golfers’ who ‘have succeeded in putting huge drives smack down the centre of familiar fairways’ ( Stafford-Clark, 1966b: 27). But this was a rare public admission: Stafford-Clark longed for the literary equivalent of an Open Championship win – to wield cultural authority having qualified as the poetic equal of Eliot, Sassoon, or Owen. These unreasonably high literary hopes defended him against a recognition of his own position within the post-war mass media and the burgeoning economy of celebrity. As his BBC career clearly shows (see Supplementary File 1), Stafford-Clark’s standing in British public life depended upon his growing celebrity, which eroded the anonymity that had, until the 1950s, been enforced upon the medical profession.

## The BBC’s psychiatrist

Stafford-Clark occasionally broadcast with British independent (i.e. commercial) television (PP/DSC/B/4/1), but his involvement with the state-funded and regulated broadcaster, the BBC, was far more sustained and is much better documented. His BBC career began at a time when the corporation’s medical broadcasters were gradually finding a more collaborative relationship with the General Medical Council, which regulated physicians, and the British Medical Association, the doctors’ trade union. Although anonymity was still demanded of physician-broadcasters, suspicion of television broadcasting was gradually waning as a new era of therapeutic optimism arrived in tandem with the greater availability of medical care via the National Health Service (NHS; Karpf, 1988: 52–53). Moreover, psychiatric and psychological expertise were specifically in demand, as there arose a public audience attuned under the NHS to a ‘new psychologized conception of primary care’ ( Hayward, 2014: 83), and confronted with ‘a flourishing of idealism about psychology potentially providing the values for a society that was fast losing them’ ( Thomson, 2006: 288). Stafford-Clark benefited from these processes, and contributed to them, forging a successful career as a media psychiatrist, broadcaster, and TV celebrity pundit.

Stafford-Clark’s BBC career began with anonymous radio appearances on the religious series, *The Silver Lining*. After a chance meeting with its producer ( Stafford-Clark, 1987: 221), Stafford-Clark was invited to test for the programme (RCONT1 letter: Rev. Richard Tatlock, DSC, 1 September 1953). The programme was based on listeners’ correspondence about their personal problems, a proportion of which required a psychiatric response. Stafford-Clark was scheduled for three weekly 15-minute broadcasts in December 1953 for a fee of 15 guineas per broadcast (RCONT1 contract: 27 November 1953). Introduced anonymously as ‘the psychiatrist’ or ‘the doctor’ (e.g. SLS 17 December 1953, cover ^2^), he covered three topics: the unwarranted stigma of mental illness; the mental patient’s ongoing spiritual sufficiency in God’s eyes; and the benefits of seeking psychiatric treatment (SLS 17 December 1953, 7). Stafford-Clark was clearly well-received, for he was quickly commissioned for another batch in January 1954 (RCONT1 contract: 6 January 1954), beginning a regular role that he continued into the 1960s.

Within two years of commencing radio work, Stafford-Clark began his TV career. In July 1955, the BBC Televisions Talks department invited him to a panel discussion on spiritual healing, in recognition of his psychiatric expertise, broadcasting experience, and Christian faith (TVART1 letter: Rev. F.H. House, Lord Bishop of Lincoln, 7 July 1955 ^2^). Stafford-Clark, though already a member of the Church of England’s Commission on Spiritual Healing, took part anonymously (TVART1 letter: Rev. F.H. House, Lord Bishop of Lincoln, 7 July 1955) at the rate of 17 guineas (TVART1 contract: DSC, 13 July 1955). He then almost immediately began semi-regular appearances as an anonymous adviser for the new half-hour evening programme *Is This Your Problem?*, in which a panel responded to viewers’ problems. A dry-run on closed-circuit TV in August 1955 (TVART1 contract: DSC, 25 August 2955) was followed by a series of broadcasts beginning September 1955 at a fee of around 20 guineas per show (TVART1 contract: DSC, 8 September 1955). Stafford-Clark consolidated this TV career by accepting *ad hoc* Television Talks invitations in the following years, thereby building a reputation for competence and availability. He took, for instance, in 1956 a fee of 8 guineas to discuss UFOs from the psychiatric point of view (TVART1 contract: DSC, 30 November 1956), and in 1960 the fee of 50 guineas plus expenses for an interview with the strip-tease impresario, Arthur Fox (TVART1 contract: DSC, 5 February 1960). Other topics include prostitution (TVART1 letter: Enid Love, DSC, 5 December 1956), misconceptions about Christianity (TVART1 letter: Rev. Oliver Hunkin, DSC, 16 May 1957), and penal reform (TVART1 contract: DSC, 18 April 1957).

Stafford-Clark’s flourishing radio and TV career led him to be unofficially designated ‘the BBC’s psychiatrist’. In 1956, the Talks Department were asked to identify a psychiatrist to discuss mescalin. They reply:

you might like […] to approach Dr Stafford-Clark who is fairly widely spoken of outside as ‘the BBC’s psychiatrist’. He […] has a very large and fashionable practice as well as many many outside engagements; but just the same I have seldom heard of his declining an invitation to broadcast. (RCONT1 memo: I.D. Benzie, Tom Wisdom, 11 March 1957)

Stafford-Clark’s status as ‘the BBC’s psychiatrist’ was sealed when, through his TV work, he met the producer Hugh Burnett, with whom he collaborated in devising the programme *Lifeline*, which ran from 1957–1962 for 56 episodes. The programme’s template was something like a clinical consultation accompanied by panel discussion of the general issues:

Stafford-Clark, appearing anonymously, will discuss a problem with a member of the public who will be appearing in vision. He will give advice to that person, show them out of the room, and then will himself move into another room to discuss the basic issues underlying the problem. At the end of the discussion he will sum up and close the programme. (T32/230 memo: Hugh Burnett, D. Knight, 16 August 1957 ^2^)

In an anonymous 1959 article for *The Listener*, Stafford-Clark explains the origins and ambitions of the programme, which clearly extended beyond a purely medical consultation. Originally entitled *What Can Be Done?*, the programme was conceived as one

which might examine not simply how, where, and by whom personal problems might reasonably be studied and help given; but beyond this might be a means of giving viewers an opportunity to think again about their own attitude to such problems in themselves and others, and above all to cultivate a greater capacity for compassion, and for an imaginative understanding of all the complexities of human life, than they had had before ( Stafford-Clark, 1959a: 568).

At Burnett’s suggestion, the programme was retitled *Lifeline* to convey not only ‘some kind of vitally needed aid, thrown out to individuals about to be submerged in a sea of trouble’, but also ‘the examination of the destiny and existence of one human being, or a group of human beings, and their repercussions on each other’ ( Stafford-Clark, 1959a: 568). Stafford-Clark explicitly frames this grander ethical vision of the programme as an intervention by psychiatry in a realm of cultural authority traditionally informed by religion. While conceding that ‘not all sincere human beings have found a religious belief to which they can give their allegiance’, Stafford-Clark asserts that agnosticism ‘is not supported by man’s own study of the intensely purposeful nature of biological processes’ ( Stafford-Clark, 1959a: 568). Situated at the intersection between biology and the psyche, ‘the psychiatrist, as a special kind of doctor, has a particular contribution to make’, for ‘[c]areful and objective study of man’s life reveals him in search not only of immediate physical satisfactions but beyond these of some ultimate point and purpose in living at all’ ( Stafford-Clark, 1959a: 568).

 Stafford-Clark thus positioned psychiatry as pre-eminently qualified to speak on ethical issues: the specialism putatively brought the dispassionate scientific authority of the biological sciences to the study of values manifested in the purposive life of the psyche. His views evidence the growing cultural authority of psychiatry – anticipating, for instance, the later statement by Carstairs in the 1962 Reith Lectures that social psychiatry could shed light on ‘marital disharmony, parental deprivation, delinquency, alcoholism, psychopathy, indeed all the indicators of social and personal disorganization’ ( Carstairs, 1963: 22), extending even to ‘our failure to comprehend the full significance of the hydrogen bomb’ ( Carstairs, 1963: 9) and the need ‘to envisage a single world government administering the affairs of mankind’ ( Carstairs, 1963: 96). In turn, Carstairs, via such pronouncements, and his editorship of the mass-market Penguin paperback series Studies in Social Pathology, clearly paved the way for R.D. Laing’s later political interventions in best sellers such as *The Politics of Experience* ( Laing, 1967; see Miller, 2015: 86–91).

Yet one must also acknowledge how, because of the autonomous logic of TV programming, *Lifeline* contributed specifically to the widening cultural authority of psychiatry. The BBC had invested in Stafford-Clark, a talented psychiatrist-presenter, and in the human and material resources necessary for a successful show. To effectively recoup their investment, the BBC indulged its star psychiatrist’s desire to extend *Lifeline*’s remit beyond narrowly medical or psychiatric issues (thereby illustrating the tendency for broadcasters to grant talented medical presenters cultural authority by presenting them as ‘Anything Authorities’ able to speak beyond their specific area of expertise ( Karpf, 1988: 112)). Episodes on topics such as ‘Mental Illness’ (12 February 1959), ‘Schizophrenia’ (9 May 1960), and ‘Schizophrenia in Children’ [i.e. autism] (30 June 1961) were indeed about psychiatry, and other episodes were health-related, such as ‘Plastic Surgery’ (3 July 1958), ‘The Battle against Leprosy’ (28 August 1958), ‘The Mongoloid Child’ (14 May 1959) and ‘Alcoholism’ (16 June 1961). But anomalous psychology and parapsychology were included via ‘The Medium’ (3 February 1960, 17 February 1960), and ‘Extra-Sensory Perception’ (2 March 1960). Other episodes were manifestly on issues of wider social and political significance: for instance, ‘Young Offenders’ (19 June 1958), ‘Children of Divorced Parents’ (18 September 1958), and ‘Corporal Punishment’ (1 January 1959). Stafford-Clark and Burnett also pursued existential or religious matters via ‘Is Religion Necessary?’ (29 October 1957), ‘Silent Order’ [on monasticism] (31 July 1958), ‘Moment of Truth’ [on near-death experiences] (6 January 1960), and ‘The Power of Faith’ (19 May 1961). Such spiritual episodes hint at Stafford-Clark’s proselytizing tendencies, which were to emerge more fully in his writing career. At any rate, the great variety of topics covered by *Lifeline* not merely indicates, but also enacts via the BBC’s programming, a growing medicalization and psychologization of British cultural life: religion, family, and the law, were all topics represented as suited to Stafford-Clark’s professional expertise by a broadcaster that was economically motivated to make the most of a star presenter and successful format.

 Indeed, the very first episode, ‘Offences Against Children’ (15 October 1957), discussed paedophilic assaults, an issue clearly as much criminological as psychiatric. The live broadcast’s format was as described by Burnett, except that (on this occasion) the panel discussion preceded the interview (T32/875 shooting script, 15 October 1957). Billed anonymously as ‘A Consultant Psychiatrist’, Stafford-Clark introduced the expert panel and led 18 minutes of discussion before conducting a 5-minute interview with an anonymous ‘Mother’ whose son had been convicted of paedophilic offences. The set dressing presented a series of social cues for the broadcast audience, providing a literal (rather than metaphorical or textual) ‘stage management’ in the performance of science advice ( Hilgartner, 2000: 12). The panel’s ‘Lounge’ is a convivial bourgeois interior with armchairs, cocktail cabinet, six-foot settee, fake bookshelves, and a fake log fire. The ‘Consulting Room’ into which the interviewee (the problem case) is temporarily invited conveys something of private medical practice, with its desk, swivel and arm chairs, (non-functioning) telephone, and framed pictures. The staging communicates a hierarchy in authority: the interviewee is segregated in time and space from the panel, and takes no part in the general discussion, which is reserved for expert colloquy in the ‘Lounge’.

 The Audience Research Report (T32/875 1 November 1957) shows a generally positive response to the first episode of *Lifeline*. The audience size was estimated at 9% of the adult UK population, and the overall reaction index was 75. In other words, the average mark for the programme was an ‘A’ – the second highest rating in the BBC’s 5-point scale running, A+ (100), A (75), B (50), C (25), C-(0) ( Silvey, 1974: 116–117)). Indeed, 25% of the sample (n=154) rated the programme an ‘A+’, and thus as exceptional, unmissable TV ( Silvey, 1974: 116). This reaction index score was ‘well above the average (66) for all talks and discussion televised during the third quarter of 1957’, so *Lifeline* was a clear comparative success. Stafford-Clark was particularly well received as both contributor and interviewer. He was viewed as ‘trusted and level-headed’, and his ‘calm competence’ was praised alongside the sagacity of his own ‘explanations and advice’. The programme was seen as reliably informative, with ‘widespread agreement […] that much valuable and, in some measure, reassuring information had emerged from the programme’. Some even wanted greater didacticism: ‘Do away with these “experts”, all arguing with each other […] and get on with the job in a straightforward teaching technique’.

*Lifeline*’s Audience Research Reports show its core audience clearly expected to be informed rather than to encounter merely light entertainment or human interest. ‘Mars and Venus speak to Earth’ (21 May 1959) interviewed George King, a supposed emissary of extra-terrestrial beings (T32/877 Audience Research Report, 3 June 1959). King, a New Age guru and founder of the still-extant Aetherius Society, claimed he ‘was able to enter into telepathic rapport with a Cosmic Master living on Venus, named Aetherius’ ( Wallis, 1974: 29), as well as with ‘other space notables – Saint Goo-Ling a member of the Great White Brotherhood living on earth, a Martian scientist, Mars Sector 6, Mars Sector 8, and Jupiter Sector 92’ ( Wallis, 1974: 30). Some viewers found King entertaining because of his absurd statements and the theatricality of his supposed trance states. However, the programme’s reaction index of 61 (closer to a B, or moderate satisfaction ( Silvey, 1974: 117)) was in fact below the average index of 71 for the previous twenty episodes of the series: most viewers thought King ‘deluded or […] an out-and-out fake’, and some thought that attention had been wasted on a ludicrous topic. Indeed, 7% of the sample (n=118) rated the programme as C-, and thus as a waste of time, very dull, highly dislikeable, and so forth ( Silvey, 1974: 117).

Nonetheless, Stafford-Clark, ever the showman, often combined public education with spectacular demonstrations that, like televised surgery ( Karpf, 1988: 54–55), exploited the possibilities of the medium. The fifth series in 1961 presented seven connected programmes on the Unconscious Mind, and elaborated an approach pioneered in the fourth series episode, ‘The Subconscious Mind’ (24 November 1959), in which Stafford-Clark and guest experts had employed hypnotic suggestion upon two subjects (LLS 24 November 1959 ^2^). Thus in ‘Reality and the Unconscious’ (LLS 24 February 1961), the hypnosis researcher Stephen Black (billed as ‘Doctor’) together with Stafford-Clark offered various instructions to four pre-hypnotised subjects – the female subject was, for instance, instructed to perceive an extra guest sitting in an empty chair, and answered questions on this imaginary visitor. Stafford-Clark concludes his spectacular authorization of psychoanalytic psychology with an exhortation to recognize the continual operation of the unconscious mind (LLS 24 February 1961). Conspicuously absent is any account or representation of psychoanalytic psychotherapy: these are stage-hypnotic demonstrations (rather than experiments) offered as proof of the unconscious mind.

Although it addressed controversial issues, *Lifeline* was rarely radical in its intent. This requirement had been clearly established by precursors such as Charles Hill, the anonymous wartime ‘Radio Doctor’, whose ‘conservatism, medical and political protected his privileged position in broadcasting’ ( Karpf, 1988: 47). The BBC’s deference to medical orthodoxy continued even as it began to plan for broadcasting on the apparently more contentious area of mental healthcare, a programming initiative motivated, as Long explains, by ‘the convergence of several factors: the desire of medical bodies to secure favourable publicity; an expansion of BBC programming on health issues; and recent developments in mental healthcare’ ( Long, 2014: 198) – the latter including new somatic methods and the increasing incorporation of psychiatry in general medicine under the NHS ( Long, 2014: 198). Although the BBC had received ‘a flood of applications from ex-patients wishing to broadcast their views’ ( Long, 2014: 198), the patient voice was firmly subordinated in the four-part 1956 series, *The Hurt Mind*, ‘the first television show broadcast in Britain devoted to mental illness’ ( Long, 2014: 194). Psychiatrists – rather than patients, or even other healthcare professionals – were the primary source of expertise for the programme. Moreover, the chosen expert consultant was none other than William Sargant, ‘a keen advocate of the new physical therapies’ and, at the time, ‘Registrar of the Royal Medico-Psychological Association’, the precursor to the Royal College of Psychiatrists ( Long, 2014: 203). Although Sargant remained off-screen, the series undoubtedly bore the impress of his somatic approach. The final programme, for instance, ‘demonstrated electroconvulsive therapy and described insulin treatment, tranquillisers, abreaction and leucotomy; the efficacy of the latter demonstrated through a conversation with a compliant leucotomised patient’ ( Long, 2014: 208). Moreover, as Long reveals, BBC documents show how, behind the scenes, there was ‘a refusal to acknowledge deviation from the sanctioned perspective of [the] series and a denial of the validity of patients’ perspectives’ ( Long, 2014: 208) – this was exemplified by the BBC’s casual dismissal of the approximately 25,000 letters it subsequently received from mental patients, including a great many ‘who had experienced shock treatment and found it frightening’ ( Long, 2014: 211).

 Although *Lifeline*’s format might have seemed to acknowledge the patient voice, the use of lay interviewees was thus more likely a response to BBC audience research on *The Hurt Mind*, which showed ‘the limitations of the didactic approach to public education favoured by many healthcare professionals’ ( Long, 2014: 211): ‘changes in attitude or knowledge tended not to occur when a point had been conveyed by a statement; confronting viewers with patients, or making the point in concrete terms, proved more efficacious’ ( Long, 2014: 211). Stafford-Clark was undoubtedly a sympathetic and effective interviewer, and his eclectic approach contrasted sharply with Sargant’s dogmatic pursuit of physical therapies. But he was equally selected as the voice of professional orthodoxy: a 1962 memo summarises Stafford-Clark’s ‘personal and professional qualities which are of great value to the BBC. He is a man with orthodox opinions on most things and can be relied on to deal with controversial subjects by sitting on the fence’ (T32/230 memo: Hugh Burnett, A.C.C.A.T. Tel, 2 February 1962). ‘The Problem of the Homosexual’ (26 November 1957), for instance, followed the sympathetic approach of Stafford-Clark’s own published views ( Stafford-Clark, 1957), so undoubtedly opposed a significant strand of public opinion. The Audience Research Report records responses varying from outright disgust – homosexuals were ‘freaks and should be presented as freaks’ – to condescending pity: the viewer who declared ‘she felt “sympathy for the afflicted now”’ (T32/875 Audience Research Report: 17 December 1957). Nonetheless, the programme was clearly perceived as educative rather than polemical – ‘giving the man-in-the-street “an opportunity to hear reliable information about the subject”’ – and was seen as a response to the Wolfenden Report, which had been published in September 1957 ( Suffee, 2016: 273) and advocated the legalization in England and Wales of homosexuality between adult males ( Wolfenden, 1957).

Generally speaking, the *Lifeline* interviewee therefore illustrated, rather than contested, expert opinion. ‘Termination of Pregnancy’ (26 February 1959) took ‘the standpoint of being firmly against abortion’, and employed an all-male panel of physicians opposed to termination, including Stafford-Clark (T32/876 memo: Head of Talks Television, DD Tel B, 24 February 1959). The female testimony came firstly from ‘a girl from the Dominions’, who ‘will say that if she had had the child it would have saved her marriage’, and secondly from ‘a girl from the North who […] was extremely ill and will tell a grim object lesson story’ (T32/876 memo: Head of Talks Television, DD Tel B, 24 February 1959). The interviewees, who were geographically and socially distant from the experts, thus validated the panel’s view. Absent were any expert female advocates of abortion, such as the Newcastle-based gynaecologist Dorothea Kerslake, who received a personal reply from editorial staff contesting her view that the programme had misrepresented the medical risks of abortion (T32/876 letter: Head of Talks Television, Dorothea Kerslake, 17 March 1959).

 But though the content of *Lifeline* was rarely controversial, it was subversive by virtue of its medium, television. Medical professionals in the UK were at the time bound by a longstanding ‘rule against “indirect advertising”, which was deemed as behaviour likely to bring the profession into dispute’, and which ‘was promulgated within the ethical guidance of the General Medical Council (GMC) and strongly supported by the British Medical Association (BMA)’ ( Loughlin, 2005: 303). Stafford-Clark was very aware that ‘[a]llowing oneself to be named in the press could and did result in accusations of unfair competition, which had the potential to elicit a hearing before the GMC for breach of ethical guidelines’ ( Loughlin, 2005: 303–304). For instance, Charles Fletcher, the presenter of BBC TV’s pioneering *Your Life in Their Hands* (1958, 1961) which depicted surgical procedures, was warned by ‘senior colleagues’ that ‘involvement with television could undermine his future career’ ( Loughlin, 2000: 179) – although, in the end, there was neither official censure nor any adverse impact on his career ( Loughlin, 2005: 304). There was no need, though, for Stafford-Clark to directly defy the edict on professional anonymity. As a September 1959 article in *The Star* tabloid newspaper reveals ( Viney, 1959), 28 episodes of *Lifeline*, alongside named media appearances in other contexts, had made him easily identifiable. *The Star*’s article responds to press coverage of the psychiatrist’s testimony in the trial of Guenther Podola, accused of murdering a police offer (and later convicted and hanged): ‘On the breakfast table in millions of homes throughout Britain today appeared the picture of Dr David Stafford-Clark, […] Director of the Psychiatric Department at Guy’s Hospital’ ( Viney, 1959); ‘But it was a face already known to a wide public as that of the anonymous doctor in BBC TV programmes such as *Lifeline*’ ( Viney, 1959). To help readers make the connection, the item included also a photograph of Stafford-Clark taken directly from the TV screen.

*The Star* article reviews the GMC and BMA edicts, and cites approvingly various doctors who criticise the code of professional anonymity, including the pioneering plastic surgeon Harold Gillies (1882–1960), who had ‘wistfully’ turned down an offer of ‘£6,500 to tell his life story in a Sunday newspaper’ ( Viney, 1959). The article’s case, though, was ultimately pragmatic rather than principled. Photography, video, and film presented a technological *force majeure*. Moreover, the article clearly implied, the medical profession ought to recognise the value of inserting media doctors into the economy of celebrity. According to Graeme Turner, ‘a public figure becomes a celebrity’ when ‘media interest in their activities is transferred from reporting on their public role (such as their specific achievement in politics or sport) to investigating the details of their private lives’ ( Turner, 2004: 8). *The Star* invites the public and medical profession to recognize that ‘[p]ublic confidence and interest in matters of medicine and health has never been higher’, precisely because of ‘the consistent efforts of the Press – and now of television – to dissolve the cold and mysterious mask of the man in the white coat and present instead the skilled human being behind it. In an age of medical marvels it is right that we should pay tribute to the men who achieve them’ ( Viney, 1959).

*The Star*’s article worked no immediate revolution in GMC policy. It was only in 1969 that the GMC’s ‘blue book’ of ethical guidance to practitioners finally and grudgingly conceded that, in the context of mass-media publishing and broadcasting, ‘the identification of a doctor need not *in itself* raise a question of advertising’ ( General Medical Council, 1969: 12). Stafford-Clark thus was billed anonymously throughout *Lifeline* until its cessation in 1962. But the anonymity edict was effectively irrelevant, as the press continued to freely name Stafford-Clark. A 1959 review in *The Spectator* of Stafford-Clark’s one-off special on Freud for independent television provides the presenter’s ‘name and job and address in full just in case the BMA’s insistence on professional anonymity should have led viewers to think they were watching a programme introduced by some quack or hack actor’ ( Forster, 1959: 18). By 1961, Stafford-Clark’s identity was taken for granted: a TV review of *Lifeline* in *The Daily Mail* complains that Stafford-Clark and his co-presenter are ‘so damned uppish’, so ‘godlike’ in their attitudes (this was one of the programme’s hypnotic spectaculars), that ‘I’d feel a lot warmer towards them if, just once, Clark hypnotised Black and told him to sing a comic song. Or Black hypnotised Clark and told him he’d forgotten to put his trousers on’ ( Black, 1961). The veil of anonymity was utterly threadbare: in Stafford-Clark’s later series *Brain and Behaviour* (1964) and *Mind and Motive* (1967), an apotropaic compliance led to the technically anonymous (i.e. nameless), but now uniquely identifying, description, ‘The Director of the Department of Psychological Medicine, Guy’s Hospital’. Moreover, in non-medical media contexts, Stafford-Clark clearly felt free to use his name, as the erosion of his anonymity, alongside his regular TV exposure, permitted him to become a part-time, but increasingly professional, TV personality. Thus when booked in 1964 onto the radio programme ‘Let’s Find Out’, he is described internally as a ‘Guest Celebrity’ (RCONT12/1 artists booking requisition, 13 July 1964), and publicly billed as ‘Author David Stafford-Clark’. This named celebrity status definitively marked his acquisition of a general cultural authority, in which his opinions on non-medical issues were counted as significant. In 1965, for instance, he contributed to *Any Questions*, the long-running (and still extant) radio precursor to BBC TV’s popular *Question Time*. In this roving show, a panel of pundits take questions on topical national issues from a local audience. Stafford-Clark was billed under his own name alongside the TV game-show panellist Lady Isobel Barnett, the poet Charles Causley, and true-crime presenter and crime novelist Edgar Lustgarten. Indeed, Stafford-Clark’s development into a professional part-time broadcaster, pundit, and celebrity was marked in 1966 by his contracting of the agents Curtis Brown Ltd (RCONT12/1 letter: DSC, Betty Proven, 17 January 1966).

Yet Stafford-Clark’s celebrity was a double-edged sword. Overexposure began to erode his value just as he was emboldened to seek greater remuneration, and to become a professional part-time broadcaster. In the first season of *Lifeline*, Stafford-Clark was paid 45 guineas per programme (TVART1 contract: DSC, 1 October 1957). But this rate was increased in 1958 to 80 guineas (TVART1 letter: DSC, R.L. Miall, 29 August 1958), and, by 1960, had reached 100 guineas per programme (TVART1 Contract: DSC, 29 January 1960). The BBC demanded exclusivity, since they did not want to lose to their commercial rivals someone recognized as ‘a good performer’ with ‘professional authority’ (TVART1 letter: Head of Talks, Television; C.P.Tel.; 29 July 1958) and even ‘star quality’ (TVART1 memo: Head of Talks, Television; C.P. Tel.; 12 November 1959). However, Stafford-Clark overplayed his hand when in 1961 he alluded to commercial invitations, and indicated he would consider abandoning his private practice for a broadcasting career if he could receive a salary of around £5,000 (TVART1 memo: Hugh Burnett, H.T.Tel, 23 March 1961). The hint at a £5,000 p.a. salary provokes alarm, and discussion begins on how, and with whom, Stafford-Clark might be replaced if he were dropped from programmes with psychiatric content (TVART1 memo: Hugh Burnett, A.H.T.Tel, 21 September 1961). The end was nigh: by 1965 Stafford-Clark was deeply angry at having been consulted on, and then excluded from, a planned major documentary, ‘The Psychiatrists’ (TVART3 memo: Donald Grattan, Editor Further Education Television, 5 April 1965). Internal correspondence explains Stafford-Clark was excluded because ‘with the amount of exposure he has had, we were frightened that if we included him, people would say he was the only psychiatrist known to the BBC’ (TVART3 memo: Aubrey Singer, Head of Outside Broadcasts Feature and Science Programmes, Television; Controller of Programmes, Television; 26 April 1965).

*Lifeline* ended in 1962, helping to clear the way for Stafford-Clark’s mass-media successors, such as Laing. While there is no documentary ‘smoking gun’, the probable reasons are clear. Stafford-Clark was expensive, difficult to handle, and overexposed. The format, after 56 episodes, was clearly reaching exhaustion. Moreover, as *The Daily Mail* reviewer indicates, the ‘uppish’ and ‘godlike’ manner of the presenter ( Viney, 1959), was out of tune with the dawning era of patient consumerism ( Karpf, 1988: 57–59). Ironically, the end of *Lifeline* left Stafford-Clark free to pitch, then devise and present seven programmes for his new series *Brain and Behaviour* (originally titled *Mind and Motive*), at the rate of 105 guineas per programme (TVART3 contract: DSC, 11 May 1964). *Brain and Behaviour*, broadcast on the new channel BBC 2, partially fulfilled Stafford-Clark’s long-standing ambition to address larger social issues, such as racial prejudice, in a discursive, essayistic format via a one-off series of his own (T32/230 memo: Hugh Burnett, A.H.T. Tel., 21 May 1959).

Archival evidence on *Brain and Behaviour* is unfortunately scant, but the successor series *Mind and Motive*, is better documented, and in fact concludes the dialectical movement of Stafford-Clark’s broadcasting career. *Mind and Motive*, reviving the previously abandoned title, was commissioned at a rate of 165 guineas per programme for eight programmes in March 1966 (TVART3 letter: Sylvia C. Hewitt, Margaret McLaren, 10 March 1966). Internal correspondence from James McCloy, the Senior Producer in Adult Education, explains his grave concerns. He notes that the proposed ‘8 personal essay or talks’ are on topics that are ‘equally the province of political science, anthropology and sociology’. In fact, they are ‘sermons’ whose ‘aim is to do good to the general public’: ‘My personal reaction is “who the devil is this dealer with sick people, to pontificate like this about the whole of human life?”. There is a curious kind of naivety about the whole project’ (T50/68 memo: James McCloy, H.F.E. Tel., 28 September 1965). As well as being confronted by escalating talent costs from their unintended monopoly supplier, who was declining in value with his increasing overexposure, the BBC also found their star psychiatrist using his celebrity power to leverage public intellectual activities that could not withstand editorial scrutiny. McCloy’s forebodings were borne out when, during the programme recordings, the BBC decided to proceed no further with two planned episodes, ‘Power and Politics’ and ‘Paying and Earning’ (cf. T50/68 ‘Mind and Motive’ proposal). The programme’s producer explains the late decision to Stafford-Clark’s agent, noting ‘the extreme brevity and vagueness of the first outline of the series’, and explaining that the remaining episodes ‘are of a far less abstract nature and draw much more on David Stafford-Clark’s clinical experience’ (TVART3 letter: Ian Martin, Margaret McLaren, 25 July 1966). This concern about Stafford-Clark’s expertise was also compounded by his preference to economize on preparation by extemporizing his broadcasts and recordings (TVART3 letter: Ian Martin, Margaret McLaren, 10 August 1966).

After the *Mind and Motive* debacle, Stafford-Clark’s radio and TV broadcasting work fell into decline, and was unofficially marked in 1973 (as he wound down his medical career) by a valedictory half-hour autobiographical radio broadcast (RCONT15 talks requisition, 9 February 1973). From the BBC’s point of view, Stafford-Clark had been a willing and capable TV performer who brought professional authority and networks, and who could generally be relied upon to eschew controversy. However, by carelessly handing him a monopoly, they found themselves beset by mounting talent costs for an increasingly overexposed performer who was unwilling to submit to editorial protocols regarding quality and preparation of content. The extent to which Stafford-Clark understood his value to the BBC remains unclear. From his point of view, the BBC gratified an ambition for public recognition, and his increasing payments were recompense for his talent, and for the risk in subverting the anonymity edict. The BBC, keen to make the most of a successful format and presenter, also indulged his ambitions for cultural authority. But, rather than admit that his celebrity was attributed by the BBC’s promotion of him as an ‘anything authority’, Stafford-Clark clung stubbornly to a belief in his qualification as a public intellectual. His account of the *Mind and Motive* story is inaccurate in light of the archival evidence, but nonetheless very revealing: ‘A searing examination of the Psychology of Prejudice and Persecution […] caused so much excitement on the BBC switchboard that the two final and equally potentially charged programmes “Power and Politics” and “Poverty and Responsibility” were cancelled by the Corporation, ending the series at six’ ( Stafford-Clark, 1987: 222). In Stafford-Clark’s fabulation, the cancellation occurs during the broadcast run, not during recording, and in response to the politically incendiary content of the programmes, rather than their deficient academic credentials. Ironically, the *Mind and Motive* cancellations gave Stafford-Clark an opportunity to claim that he had been, in effect, censored by the Establishment.

## Dictating to the public

Stafford-Clark’s involvement with the BBC parallels to a large extent his writing career, which is also marked by the pursuit of opportunities to exercise cultural authority, accompanied by intellectual over-extension, declining credibility, and self-righteous conflict with editorial protocols. McCloy, of BBC Adult Education, perceptively noted (above) Stafford-Clark’s urge to deliver ‘sermons’ whose ‘aim is to do good to the general public’. The evidence from Stafford-Clark’s print output (see Supplementary File 2) amplifies this insight by revealing the spiritual biography that putatively authorized his non-psychiatric statements: Stafford-Clark was on a mission to dictate his spiritually authorized insights via the mass-media, and to strategically exploit the publishing opportunities opened up by his psychiatric celebrity.

Stafford-Clark’s writing career expresses the ambition, manifest also in his broadcasting, to comment on all manner of issues, even where the psychiatric perspective is partial or even irrelevant, and where other sources of expert knowledge might claim authority. Stafford-Clark’s views on homosexuality, for instance, were frank, liberal, and consistent: ‘nothing but arrogant hypocrisy, partiality, and cynicism can support the present legal discrimination against male homosexuality’ ( Stafford-Clark, 1964a: 60). His 1957 article ‘Homosexuality’ (based on an earlier lecture to the Medico-Legal Society ( Stafford-Clark, 1957: 65n)) surveys anthropological and historical sources, discusses the contemporary legal situation in the United Kingdom, explores medical and sexological accounts of the causes and frequency of male homosexuality, and presents ethnographic testimony from Stafford-Clark’s acquaintance with ‘homosexual London’ ( Stafford-Clark, 1957: 73). The psychiatrist repudiates the medicalization and clinical treatment of homosexuality, stating that ‘homosexuality is not curable by psychotherapy’, and argues that therapy can work mainly toward self-acceptance ( Stafford-Clark, 1957: 76). The Wolfenden Commission (to which Stafford-Clark briefly alludes ( Stafford-Clark, 1957: 76–77)) were to agree when they finally published their report ( Wolfenden, 1957) in the same year (to which Stafford-Clark responded with a sympathetic edition of *Lifeline* (see above)). Stafford-Clark’s statements on homosexuality certainly deployed a great deal of psychiatric expertise, and clearly brought professional risks (including potentially career-threatening gossip that Stafford-Clark was himself a closeted homosexual (PP/DSC/E/1 letter: DSC, Dr Richard Fox, 2 October 1963)). However, they also extended well beyond his clinical expertise: in ‘Homosexuality’, Stafford-Clark mobilizes a fundamentally rights-based argument, while also discussing the legal history of homosexuality, the inconsistencies in the policing of sexual mores, and the penological complexities of criminalization – at one point, he remarks ‘I should remind myself that I am a doctor, and not a lawyer’ ( Stafford-Clark, 1957: 76).

Stafford-Clark’s consistent, and prescient, efforts to depathologize homosexuality were among his most laudable contributions to wider societal and cultural debates, and rank alongside his wartime challenge to the RAF’s LMF quasi-diagnosis. His work on prejudice, while praiseworthy in its underlying spirit, is more equivocal. Stafford-Clark was among the British psychiatric and psychological cultural authorities who resisted racism and racist attitudes, but who in doing so pathologized racial prejudice. The template for the post-war pathologization of racism had been set out in the USA, where Frankfurt School intellectuals, such as Theodor Adorno, were dissecting the racial attitudes of the defeated Nazi culture. The publication in 1950 of *The Authoritarian Personality* ( Adorno *et al*., 1950), a treatise that attempted to ‘frame racism as a psychopathological problem’ ( Thomas & Byrd, 2016: 184), stimulated a growing and influential body of research and practice: ‘The notion that a sick society produces sick individuals would be a recurrent theme within mental health discourse through the 1950s and, consequently, would find itself within the claims of public officials and activists who drew upon the authority of medical and psychological science to make claims about the nature, and consequences, of extreme racism’ ( Thomas & Byrd, 2016: 184). Stafford-Clark pursued this agenda in his own broadcasting and publishing, and throughout his career recycled ideas aired in his 1960 Robert Waley Cohen Memorial Lecture, *The Psychology of Persecution and Prejudice* ( Stafford-Clark, 1960), and continued in such publications as ‘The Psychology of Prejudice and Persecution’ ( Stafford-Clark, 1961b), and ‘The Psychology of Prejudice’ ( Stafford-Clark, 1968). Stafford-Clark’s views on racial prejudice show a peculiar mingling of both psychological (specifically, psychoanalytic) and Christian motifs. Stafford-Clark entirely dismisses sociological accounts of racial prejudice ( Stafford-Clark, 1968: 78), and offers instead a ‘Christian correction’: ‘the various keys to prejudice all hang from one ring, […] the innate, inevitable, yet tragic self-centredness of the human personality’ ( Stafford-Clark, 1968: 78). Prejudice and bigotry are acquired only because of the ‘prideful and instinctively self-willed aggressive aspect of each one of us’ ( Stafford-Clark, 1968: 77): ‘When we cannot achieve what we want, when we are disappointed, when our hopes outrun our attainments, when somebody else gets the job, or the girl, or the money, we would like to be able to tell ourselves that it wasn’t our fault; that they had an unfair advantage’ ( Stafford-Clark, 1968: 77). This is explicitly a story of psychologically refurbished ‘original sin’ ( Stafford-Clark, 1968: 86), ‘the inescapable self-centredness, separateness, and tragic personal pride of each individual one of us’ ( Stafford-Clark, 1968: 88).

 This peculiar repristination of Christian narrative patterns was by no means confined to Stafford-Clark. Carstairs also offered a roughly simultaneous attempt to depict the analysis of racial and national prejudice as a realm in which psychology was the rational inheritor of intuitions previously couched in the language of theology:

Religion talks in terms of guilt, and of the way in which the seven deadly sins obscure our vision of God’s purpose for mankind. Psychiatry also deals with guilt and with the conflicts in our own personalities which prevent our seeing things clearly. Both the religious and psychiatric interpretations of our present predicament suggest we shall only be freed from fear of each other when we recognize, and abate, our own destructive impulses. An involuntary bias of this kind often has its origin in experiences of childhood ( Carstairs, 1963: 96–97).

Carstairs’ solution was, like Stafford-Clark’s, oriented toward individual psychological health: ‘We have made progress in reducing the amount of severe poverty in our society: our next task is to try to ensure that children are not deprived of the emotional sustenance which they need in order to develop into well-balanced beings’ ( Carstairs, 1963: 97).

Such mingling of religious declarations with psychological analysis could, however, present problems for peer-review protocols. Miller, for instance, notes how Stafford-Clark’s ‘religious proclamations’ in *Psychiatry To-day* led to disputes with Penguin, who would rather such material was excluded from their layperson’s guide to psychiatry ( Miller, 2015: 83). This tendency to preach, noted also by the BBC, hints at a missiological ambition in Stafford-Clark’s public intellectual ambitions: his account of racial prejudice as original sin was spared academic scrutiny, but still gained significant circulation in pamphlets, non-expert periodicals, lectures, and broadcasting (via *Brain and Behaviour* and *Mind and Motive* episodes, for instance). Because of his celebrity status, Stafford-Clark had a disproportionate access to publishing opportunities, and fully exploited the compositional technique that had served him well for *Psychiatry To-day*. His prose texts (although presumably not his poetry) are essentially revised versions of extemporised lectures that have first been transcribed – and no doubt lightly edited – by a secretary. In personal correspondence, Stafford-Clark reveals his preference simply to dictate a rough draft to his secretary, as he might with normal medical correspondence: ‘in order to maintain output […], I find a dictaphone and a faithful, competent and efficient secretary quite indispensable’: ‘although I do a great deal of correction of typescript for published work, I still produce it all in the raw by dictation. Virtually every word of the Pelican book was dictated, and practically every piece of published work since then was similarly produced’ (PP/DSC/E/1 letter: DSC, Tom Angus, 17 February 1956).

Stafford-Clark’s original discourse was effectively a spoken lecture, or indeed sermon, and thus there are occasions when the deficiencies of this improvised orality become clear, particularly where he was no longer on familiar psychiatric ground. For instance, in a 1969 contribution on capital punishment for a volume entitled *The Hanging Question*, Stafford-Clark lends his voice to a group that includes clergy, academics, novelists, journalists, and two other psychiatrists. In an unintentionally revealing statement, Stafford-Clark begins his chapter by complaining about the commissioning process: ‘a length of up to three thousand words, a deadline by six weeks from 23rd May, and no fee’ ( Stafford-Clark, 1969: 127) – a timescale which means that ‘I am currently working at Guy’s and I dictate this letter on tape over the Whitsun weekend’ ( Stafford-Clark, 1969: 132). Predictably, his ethical and legal analysis is trivial: ‘The real question is not whether capital punishment is justifiable, because the answer is simple: it is not. Judicial murder is murder no less than casual or criminal murder’ ( Stafford-Clark, 1969: 130). This is no argument, merely question-begging assertion. Stafford-Clark then offers his alternative: he rejects penal incarceration, and instead proposes a life sentence served on ‘an island, populated only by people convicted of murder and those who are paid to see that they remain there peacefully and do not return to the society from which they have been exiled’ ( Stafford-Clark, 1969: 130). Without any intended irony, Stafford-Clark then proposes, in effect, a *de facto* return of the death penalty, since the island’s warders ‘should be armed, and armed if necessary to kill’ ( Stafford-Clark, 1969: 131). The essay is little better than an off-the-cuff peroration, and shows precisely the extent to which Stafford-Clark’s sermonizing propensities eroded his intellectual credibility, and exploited instead his dwindling celebrity.

 The question arises of why Stafford-Clark was so willing to step beyond the boundaries of his psychiatric expertise. As will be shown (below), he generally professed a high estimation of his own abilities. However, there was also an additional element of spiritual authorization in his activities: Stafford-Clark was frequently speaking from a position of faith rather than of academic or medical expertise. In a 1962 contribution to an essay collection based on religious radio broadcasts for high-school students, Stafford-Clark explains his views on ‘Suffering and Character’: ‘I, as a doctor, am forced to see that not only is life beautiful and exciting and wonderful and real, but also that life is cruel and wasteful. And sometimes we have to accept the waste and cruelty as part of an infinite process that we can never wholly understand’ ( Stafford-Clark, 1962: 110). By accepting this mystery,

even the worst kinds of suffering can acquire a transcendent significance […]. I have seen men and women, apparently without anything at all inside them to meet the disasters which confronted them – the personal, the physical, the emotional, the moral disasters – who seemed to receive something from beyond themselves. They seemed at the moment of crisis to be utterly transformed by courage ( Stafford-Clark, 1962: 110).

While Stafford-Clark may indeed have witnessed this transformation, he was also testifying to his own conversion experience. In his contribution to *Dialogue with Doubt* (1967), based on religious dialogues broadcast on independent television, Stafford-Clark describes how he ‘became an agnostic drifting towards atheism’ ( Stafford-Clark & Comfort, 1967: 122) until the death of his brother in the war, which occasioned ‘a kind of minor road to Damascus affair’ ( Stafford-Clark & Comfort, 1967: 122). In this turning point, Stafford-Clark receives something from beyond himself:

The fact that my brother had been killed […] shattered me completely and I went to my room and wept quietly. […] I thought, ‘Well, I haven’t believed in this God business for a hell of a long time, and this is God’s chance, as it were’. I needed some help from somewhere […] to be able to stand in front of people not just crying. So I put it up to God […], ‘If there’s anything there, give me the guts or the presence. Give me the power to control myself’, which I knew I had not got. ( Stafford-Clark & Comfort, 1967: 123)

‘Suffering and Character’ therefore conveys abstractly Stafford-Clark’s own experience of a transformative spiritual regeneration: ‘I see this transcendence, this shining, overcoming of the worst kinds of bitterness, waste, and cruelty in people who turn openly and say, “I can’t do this on my own, but with Christ’s help and love, I believe I can” […]. This is an idea which under all and any circumstances can still today […] work miracles’ ( Stafford-Clark, 1962: 111).

Callum Brown argues that British secularization was ‘a remarkably sudden and culturally violent event’ rather than a ‘long, inevitable religious decline’ ( Brown, 2009: 175–176). The crucial change was a cultural revolution in which ‘piety “lost” its discursive home within femininity. […] “the angel in the house” to use an historian’s cliché, was now negotiable and challenged discursive terrain’ ( Brown, 2009: 179). Miller contends that Laing’s life and work shows an attempt to preserve such ‘discursive Christianity’, even as organizational forms declined, so that Laing himself took on the previously feminized role of the guardian of piety ( Miller, 2012: 141). Stafford-Clark clearly has a similar motivation to preserve and rework Christian narratives, whether in his cultural sermons (racial prejudice as original sin), or in the autobiographical narrative that underlies them, ‘a life-journey, using notions of progression, improvement and personal salvation’ ( Brown, 2009: 185). This tacit spiritual autobiography underlies religious pronouncements, such as Stafford-Clark’s 1956 book chapter for the collection *Christian Essays in Psychiatry*. He insists there is a universal religious need: ‘careful and objective study of man’s life reveals him in search […] of some sort of point and purpose in living at all. This search […] is an inescapable aspect of human existence’ ( Stafford-Clark, 1956: 14). As a theological, philosophical, or even psychological exercise, Stafford-Clark’s chapter is lacking, with very little by way of empirical or philosophical argument for his thesis, which he insists is simply ‘an inescapable fact’ ( Stafford-Clark, 1956: 18). The persuasive force comes from the text’s laconic, confident assertions about what is essential, universal, and inescapable, in its extrapolation from the *ex cathedra* statement: ‘Man is not, and cannot be, content to accept life as meaningless’ ( Stafford-Clark, 1956: 13). The biographical authorization by faith extends to a quasi-psychoanalytic assertion that ‘the attempt to deny it [i.e. religious need] inevitably leads to an even more violent assertion of the natural demand, most of all in the minds of those who have neither acknowledged it nor consciously sought its fulfilment’ ( Stafford-Clark, 1956: 16). Those who feel no ‘need to believe’ are self-deceived, and what is required, presumably, is spiritual self-clarification and inner transformation. Just as Freud discovered the secret of infantile sexuality, so Stafford-Clark knows from his own life the necessity of religious belief. Psychiatry thus has an adjunct role in the promulgation of Christianity (and presumably of a Christian society), for ‘any success achieved by psychiatry in straightening out a tangled mind, in helping a man to think more clearly and honestly, must inevitably help him also to open his mind and his heart to God – if he so chooses’ ( Stafford-Clark, 1956: 23).

 Understanding Stafford-Clark’s spiritual autobiography clarifies his statements on sex and sexuality, which go far beyond the psychiatric and legal discourses that underpin his statements on homosexuality. In Gillian Freeman’s 1967 book *The Undergrowth of Literature*, Stafford-Clark supplies ‘a foreword for a book dealing with the literature of sexual fantasy, and particularly with the fantasy literature of sexual perversion’ ( Stafford-Clark, 1967a: xi). Stafford-Clark, in an echo of his views on psychiatric popularization (above), employs a sweeping rhetoric of colonial enlightenment: ‘only by bringing into the open light of day the tangled undergrowth of literature, which swarms with its own lonely intensity and vigour, like the twilight vegetation of a sub-tropical jungle where the sun never penetrates, can we begin ourselves to understand […] the meaning of this undergrowth, and the perversions to which it is dedicated’ ( Stafford-Clark, 1967a: xxv). Stafford-Clark’s moralizing about sadomasochism, bondage, and fetishism is emphasized by his condescending allocation to Freeman of ‘the intuitive wisdom so often granted to women’ ( Stafford-Clark, 1967a: xxv): she has ‘taken a certain refuge in flippancy’ because ‘as a woman as well as a writer seeking to find love […], she would have recoiled in disgust had she not been able to retain a certain salutary detachment’ ( Stafford-Clark, 1967a: xxiv). Freeman is ushered into a novel variation on a familiar role: the woman’s place is not the ‘angel in the house’ but rather, one might say, ‘the angel in the bed’, who should recoil instinctively at the loss in ‘sexual perversion’ of ‘any kind of relationship with another human being having to do with love or tenderness, or even with their existence at all’ ( Stafford-Clark, 1967a: xvi). The same gendering of sacred sexuality appears also in Stafford-Clark’s 1967 *Sunday Times* opinion piece on the Olympia Press’s erotica, where he speculates that Pauline Réage, the author of *The Story of O*, was in fact ‘a male masochistic homosexual transvestite’ ( Stafford-Clark, 1967b: 34), rather than (as we now know) the pen-name of Dominique Aury, born Anne Desclos ( Wyngaard, 2015: 980). The spiritual and transcendental aspects of monogamous, romantic heterosexuality are further elaborated in Stafford-Clark’s anonymous 1966 article on ‘Frigidity’ for the pioneering woman’s magazine *Nova*. Stafford-Clark states that ‘[t]his is a personal and not a medical article. It offers no direct advice and makes no pretensions to solving personal problems by generalized impersonal counselling. Its aim is to convey an aspect of sexual truth’ ( Stafford-Clark, 1966a: 56). In fact, there is a great deal of impersonal counselling and advice in the article’s anatomical demystifications and debunking of sexual myths, but the ‘aspect of sexual truth’ appears in Stafford-Clark’s frankly sanctifying descriptions of ‘the mystery of sexual love’ ( Stafford-Clark, 1966a: 58), ‘the most transcendent, ecstatic, and liberating physical and emotional experience of which human beings together are capable’ ( Stafford-Clark, 1966a: 58).

 Stafford-Clark’s heterodox sermons on sex and sexuality, anonymous or otherwise, were clearly conceived by their author as in some way separate from his psychiatric writings. Yet he could not simply construct a discrete literary persona. A reader’s letter in response to another (non-anonymous) *Nova* article ‘A Consumer’s Guide to the Do-it-yourself Sex Books’ ( Stafford-Clark, 1966b: 22) explains that she is ‘rather disappointed in your article in July’s *Nova*, I think you are running the risk of becoming a Godfrey Winn of Psychiatry’ (PP/DSC/E/1 letter: anonymized correspondent, DSC, 1 July 1966). During the 1960s, Winn (1906–1971), who had abandoned an earlier career as a second-rate novelist, was a columnist with a largely female following who ‘wrote most regularly for the *Daily Mail*, *Woman*, and *Woman and Home*’, and was also ‘a fluent and popular television personality’, who had been ‘chosen to anchor some of the first series on Independent Television, including *As Others See Us*, in which he considered the personal problems of viewers, and *Godfrey Winn Speaking Personally*’ ( Bingham, 2014). Stafford-Clark was also a television personality, and at times nearly an agony uncle himself, so the letter seems to have touched a nerve. He replies at some length, with the trenchant statement that his ‘writing can be divided quite clearly into that which has to do with the practice of medicine, and that which has to do with the acceptance and fulfilment of commissions and opportunities which I am careful to keep entirely separate from my writing, teaching, and indeed all other activities connected in any way with my patients’ (PP/DSC/E/1 letter: DSC, anonymized correspondent, 4 July 1966). The *Nova* article, written ‘partly for fun, partly for money’ (PP/DSC/E/1 letter: DSC to anonymized correspondent, 4 July 1966) is thus, allegedly, the product of a separate career as a professional author, rather than a work that flows from his media psychiatry. Stafford-Clark’s response to the correspondent demonstrates an insecurity about his cultural authority. He recognizes that his *Nova* article lacks psychiatric authorization, so he offers instead a literary career as qualifying activity. Yet the tag, ‘a Godfrey Winn of Psychiatry’ was essentially valid: Stafford-Clark could deliver his homilies precisely because he was a skilled radio and TV broadcaster perceived publically as offering psychiatric and medical expertise.

Stafford-Clark’s relationship with *Nova* soon ended, for the magazine wanted psychiatric rather than literary or spiritual significance in its author. In typically trenchant fashion, Stafford-Clark is drawn into a dispute over a spiked article on ‘Violence as a Personal Phenomenon’ (‘I’m still quivering with inward fury, outrage, protest, and indignation’ he declares in a letter of complaint, having been informed that *Nova* ‘“don’t consider that it is up to my usual standard”’ (PP/DSC/B/3/9-2 letter: DSC, Dennis W. Hackett, 3 March 1967)). The rejection criticises the article for ‘stereotyped thinking’ and ‘non sequitur’ reasoning, (PP/DSC/B/3/9-2 letter: Michael Wynn Jones, DSC, 1 March 1967), and the episode ends (along with any hope of future commissions from *Nova*), when Stafford-Clark gets his fee, although not publication (PP/DSC/B/3/9-2 letter: Dennis W. Hackett, DSC, 14 March 1967). Stafford-Clark’s disciplinary limitations were exposed further in *Five Questions in Search of Answer*, the companion volume to his 1970 Nelson Lectures at Lancaster University. These lectures, under the auspices of the Department of Religious Studies, and sponsored and published by the Edinburgh-based Christian publishers Thomas Nelson ( Stafford-Clark, 1970: 139), were Stafford-Clark’s opportunity to present a summative monograph (‘written before, and deliberately independently from, the lectures’ ( Stafford-Clark, 1970: xi)), drawing together ideas that ‘have taken half a lifetime to accumulate’ ( Stafford-Clark, 1970: xi). As well as giving Stafford-Clark a further opportunity to circulate his poetry (see above), *Five Questions* also became an avenue for bottom-drawer musings, such as his Appendix 1 on ‘Violence’, which re-uses the spiked 1967 *Nova* article on the same topic. The main text of the book also substantially re-uses earlier material: the second chapter, for instance, involves lengthy *verbatim* unacknowledged re-use of Stafford Clark’s chapter on ‘The Nature of the Problem’ ( Stafford-Clark, 1956), contributed to *Christian Essays in Psychiatry*. In the epilogue to *Five Questions*, Ninian Smart, the distinguished pioneer in religious studies at Lancaster University, explains though that although the book’s ‘principal concern is with the psychology of religion’ ( Stafford-Clark, 1970: 174), its author has ‘adopted an imaginative, poetic approach’, which ‘moves forward from one image and argument to another’ ( Stafford-Clark, 1970: 175). This ‘obliqueness’ ( Stafford-Clark, 1970: 175) means it would be ‘presumptuous’ of Smart ‘to attempt to present Dr Stafford-Clark’s main argument’ ( Stafford-Clark, 1970: 176). The book, in other words, is a patchwork lacking a clear argument or overall thesis. Indeed, the BBC agreed: having earlier arranged to record the Nelson Lectures (RCONT12/2 letter: Dr Archie Clow, DSC, 31 March 1970), they concluded that there was no potential for a series of broadcasts or even a single programme (RCONT12/2 letter: Dr Archie Clow, DSC, 18 May 1970). A positive review came from the theologian David Cairns, who, while unable to find an argument in at least one chapter, was impressed by the explicit biographical authorization: ‘This book […] is the story of one man’s struggle for Christian faith in the face of remorseless pressure of the problem of evil. Written with utter honesty, it draws on the agonising experiences of a doctor, and a man who has faced some of the most harrowing and challenging experiences of war’ ( Cairns, 1974: 102).

 Nelson had commissioned *Five Questions* on the basis of ‘a brief statement of the author’s aims and intentions’ ( Stafford-Clark, 1970: 139), but other publishers were to prove cannier in their assessment of Stafford-Clark’s public-intellectual aspirations. In 1977, Stafford-Clark was invited to deliver the prestigious Gifford Lectures at the University of Saint Andrews. Eventually titled ‘Myth, Magic, and Denial: The Treacherous Allies’, these were delivered in January-February 1978 (PP/DSC/C/2 Gifford Programme), with Stafford-Clark extemporising from a series of handwritten ‘working notes’ covering a few sides of notepaper (PP/DSC/C/2 ‘Working Notes‘). He addressed familiar themes from his career such as ‘Myths of Racial Supremacy’, and seems, in a lecture such as ‘Myths of the Market Place’, to include ideas from the cancelled *Mind and Motive* programmes of the 1960s (PP/DSC/C/2 Gifford Programme). Such minimal textual preparation – which Stafford-Clark had honed as a TV presenter – was, however, inimical to his hopes of a published volume. Scottish Academic Press prudently insists on peer-review of a completed final text, before committing to publication (PP/DSC/C/2 letter: Douglas Grant, DSC, 6 June 1978). Stafford-Clark replies that his own standing is sufficient to guarantee the quality of the eventual written text: ‘The lectures themselves […], the University’s own decision to request their publications, and my own willingness to write a clear concise updated version […], up to the highest standard of my established literary reputation, provide all the grounds necessary for your “consideration”’ (PP/DSC/C/2 draft letter: DSC, Douglas Grant, 14 July 1978). Scottish Academic Press, naturally, were unmoved by Stafford-Clark’s self-estimation, and declined to proceed further (PP/DSC/C/2 letter: Douglas Grant, DSC, 28 July 1978).

## Conclusions

This article began with a comparison between Laing and Stafford-Clark: both were media-friendly psychiatrists who became celebrities (internationally for Laing, nationally for Stafford-Clark). Both used access to media channels facilitated by their ‘star quality’ to exercise a general cultural authority, and each shared a similar ambition to be of literary and cultural significance (c.f. Beveridge, 2011: 38–44). As their careers matured, each dwindled in celebrity, and the quality of their work also declined, with opportunistic commissions, recycling of ideas, dissemination of bottom-drawer material, and conflict with peer-review protocols (c.f. Miller, 2015: 86–91). Notably, the same trajectory obtained despite their widely different relations to the psychiatric mainstream. Laing was prominent amongst so-called ‘anti-psychiatrists’, whereas Stafford-Clark’s psychiatric philosophy was encapsulated neatly by the BBC, who described him as ‘a man with orthodox opinions on most things’, who ‘can be relied on to deal with controversial subjects by sitting on the fence’. What Laing and Stafford-Clark shared was the exercise of cultural authority in an era in which psychotherapies, broadly conceived, had acquired a particular existential significance. Nikolas Rose traces the post-war cultural authority of the psychological disciplines to a societal shift in which the self ‘is obliged to construe a life in terms of its choices, its powers, and its values. Individuals are expected to construe the course of their life as the outcome of such choices, and to account for their lives in terms of the reasons for those choices’ ( Rose, 1990: 227). The space for a more autonomous, self-directed and examined life was opened up, in the UK, when ‘in the late 1950s and the 1960s a fundamental shift in political rationality began to occur’ ( Rose, 1990: 224): ‘A “private” realm of personal desires and predilections was to be delineated, to be regulated by the force of public opinion, by the pressures of civil society and personal conscience, but not by the use of the coercive powers of the state’ ( Rose, 1990: 225). Rose cites, for instance, the Wolfenden Report as epitomising a ‘welter of reforming legislation that reconstructed the modes of control over the moral conduct of citizens: prostitution, homosexuality, obscenity, alcohol consumption, betting and gaming, censorship in the theatre, abortion and divorce’ ( Rose, 1990: 225).

 Stafford-Clark’s career illustrates, at least in part, Rose’s statement that ‘the rationale of psychotherapies – and this applies equally to contemporary psychiatry – is to restore to individuals the capacity to function as autonomous beings in the contractual society of the self’ ( Rose, 1990: 227–228). The psychiatrist’s statements on homosexuality, for instance, firmly depathologized (and decriminalized) this sexual preference, offering psychotherapy as a means to overcome neurotic guilt over sexual orientation. But the figure of the celebrity psychiatrist goes beyond merely the promotion of psychotherapeutic technologies in the ‘society of the self’. Rose states that

the same forces that de-legitimate ‘public’ interference in ‘private’ life open the details of wishes, desires, and pleasures to a plethora of new regulatory forms, no less powerful for being ‘decoupled’ from the authoritative prescriptions of the public powers. Television, advertising, magazines, newspapers, shop windows – the signs and the images of the good life were inscribed on every surface that could carry their imprint ( Rose, 1990: 225).

Stafford-Clark could serve ‘double duty’ by promoting psychotherapeutic solutions in the private sphere, while also exploiting media access in order to positively shape opinions about the good life. The latter activity was particularly marked with regard to the significance of religion, which was increasingly a matter of private commitment rather than state-sanctioned obligation. Due weight should therefore be given to the importance of ‘para-social’ factors in Stafford-Clark’s career as a public figure. The term ‘para-social’ acknowledges that, amongst the uses and gratifications of broadcast media, is the opportunity to ‘form relationships with media characters, albeit unilateral relationships, that affect us in ways that resemble any other relationship with a person’ ( Giles, 2000: 62). In its original usage, the term was orientated toward debunking ‘the illusion of face-to-face relationship’ ( Horton & Wohl, 1956: 215): the mass-media audience are seen as ushered into a compliant and mystifying social role that complements the illusion of intimacy offered by the so-called ‘persona’ of a skilled broadcaster ( Horton & Wohl, 1956: 216–219). In a more charitable, phenomenological model, however, the para-social (or simple ‘sociable’) relationship expresses the fundamental truth that ‘[t]he relationship between broadcasters, listeners and viewers is an *unforced* relationship because it is unenforceable. Broadcasters must, before all else, always consider how they shall talk to people who have no particular reason, purpose or intention for turning on the radio or television set’ ( Scannell, 1996: 23). Radio and television address mass audiences not as a crowd but as individuals, which ‘means more than chatty mannerisms and a personalized idiom (“I”, “you” and “we”). It means orienting to the normative values of ordinary talk in which participants have equal status and equal discursive rights. In short, it is no use talking *at* listeners, or talking *down* to them, for if you do they can simply switch you off’ ( Scannell, 1996: 24).

Admittedly, the TV reviewer, who criticises in 1959 Stafford-Clark’s ‘uppish’, ‘godlike’ manner, shows that that while the psychiatrist may have had the knack of addressing viewers as individuals, he apparently had some difficulty in adopting the less didactic manner needed to talk ‘to’ listeners, rather than ‘down to’ them. Nonetheless, Stafford-Clark rose to prominence because he was willing and able to meet the para-social requirements of the mass media. The ‘Lounge’ in *Lifeline*, for instance, was a sociable, domestic space, which the audience were invited to share with Stafford-Clark and his various discussants. Moreover, the para-social relationship mandated by contemporary celebrity is evidenced by *The Star*’s aspiration, amenable to Stafford-Clark, that media coverage should ‘dissolve the cold and mysterious mask of the man in the white coat and present instead the skilled human being behind it’. Stafford-Clark was thus in the vanguard of physicians who, with journalistic assistance, eroded professional edicts on anonymity, and encouraged medical organizations to exploit the mass media, and to work with, rather than against, the para-social logic of celebrity. (Indeed, Stafford-Clark clearly paved the way for Laing’s career a few year later, for the Scottish psychiatrist had no qualms about identifiability, despite the GMC guidance on anonymity and indirect advertising.) Stafford-Clark’s capacity for apparent unforced intimacy with viewers and listeners undoubtedly abetted his exercise of cultural authority. As much as he might have hoped for intellectual leadership, his views on the good life had currency because the new broadcast media brought him, like presenters such as Godfrey Winn, into a phenomenological proximity with households across the United Kingdom. The Stafford-Clark who appeared in one’s social circle, as if only a few feet away on the TV screen, might offer, for instance, an exemplary spiritual biography, like that retold on ITV’s *Dialogue on Doubt* in 1967, or expatiate on any number of *Lifeline*’s wider topics, addressing ‘the destiny and existence of one human being, or a group of human beings, and their repercussions on each other’. The dearth of audio-visual material makes direct exploration of Stafford-Clark’s para-social persona rather difficult, but the significance of this development in broadcasting might be more fully explored in celebrity psychiatrists for whom there is significant material available. The roster includes Laing, of course, but also extends to more recent figures, such as Anthony Clare (1942–2007), presenter of the BBC radio programme *In the Psychiatrist’s Chair* from 1982 to 2001 ( Nolan, 2008).

What worked in the intimate, conversational format of *Lifeline* or similar programmes, fared less well, however, in other regions of Stafford-Clark’s oeuvre. The discursive, essayistic format demanded by a series such as *Mind and Motive* – or in the production of quality journalism, quasi-academic essays and monographs – could not easily accommodate Stafford-Clark’s exercise of cultural authority as a celebrity: in particular, his efforts to renew and continue the narratives of discursive Christianity – via prejudice as original sin, or through espousals of sacred sexuality – came across merely as sermonising, rather than as intellectually well-founded. Despite his apparently greater psychological wellbeing, Stafford-Clark’s overall career therefore shows the same pattern of diminishing returns as Laing’s. The cultural economy of celebrity offers a de-psychologized explanation for Stafford-Clark’s rise and fall (with corresponding implications for our understanding of Laing, and perhaps the far more recent case of TV psychiatrist Raj Persaud’s three-month suspension for plagiarism in 2008 ( Dyer, 2008)). Stafford-Clark was akin to those highly successful media intellectuals who ‘most need to be reminded that a condition of their status is the continued perception that they are also doing work in a specialized sphere which measures up to the highest standards of that specialism’ ( Collini, 2006: 486). Without this continued perception of qualification, such figures ‘will either have to become full-time media celebrities, or they will find that the dry rot of repetition and overexposure does its deadly work and that the invitations to pronounce become rarer and rarer’ ( Collini, 2006: 486). Stafford-Clark’s temporary monopoly on TV psychiatry facilitated his overexposure in the mass media, both print and broadcast. His value declined, and he responded by seeking further opportunities precisely when he should have been restricting his exposure, and replenishing his intellectual capital (the contrasting career of Carstairs, whose media appearances were much more infrequent, would have been a better model to emulate). Moreover, Stafford-Clark’s costs remained high, since he demanded recompense for professional risks, and for the opportunity costs against public or private practice; his editorial relations weakened, for overexposure meant he was stretched too far intellectually; and his preference for the ease of oral composition by dictation merely weakened the quality of his work. The *Nova* reader, who in 1966 complains that Stafford-Clark, by writing personally revealing articles on sex manuals, has become the ‘Godfrey Winn’ of psychiatry, is warning against overexposure and brand dilution, and against the risks of the para-social persona. Stafford-Clark, though, never fully grasped the reality of his predicament, and preferred instead to indulge in defensive rationalizations, such as those expressed in his autobiographical reflections in 1987, where he laments changes in television broadcasting towards the late 1960s, by which ‘[l]ive television was virtually confined to game shows and sports; while any kind of “serious” subject was extensively prerecorded on film or video, and subsequently edited to the overall length, balance, and proportions envisaged by the producer, but not necessarily approved or controlled by the presenter’ ( Stafford-Clark, 1987: 221). This supposed ‘journalistic takeover of TV’ (PP/DSC/B/4/1) was part of a general pattern where Stafford-Clark projected blame onto a series of outsiders to the medical profession – be it journalists, editors, or the lay public – rather than recognizing that the allure of celebrity had drawn him into an economic life cycle over which he had little control so long as he continued to pursue broadcasting and publishing opportunities.

## Footnotes

^1^ **Wellcome Library:** Stafford-Clark’s Personal Papers (ten boxes) are held at the Wellcome Library, 183 Euston Road, London, NW1 2BE. These are filed under the reference PP/DSC, and are arranged as follows: A Personal; B Broadcasting and Publishing; C Lectures; D Membership of Professional Societies; E Correspondence.

In-text references are in parentheses, and are in the form (PP/DSC/A/1 …) etc. The Wellcome file reference is followed by specifying information, such as the type of material (e.g. letter, memo) followed by (where available and appropriate) name of originator, name of recipient, date.

^2^ **BBC Written Archives Centre:** Unfortunately, there appears to be no readily accessible audio and audio-visual material relating to Stafford-Clark’s radio and TV broadcasting. However, there is substantial archival material relating to Stafford-Clark’s BBC career held by BBC Written Archives, Caversham Park, Peppard Road, Reading, RG4 8TZ.

Contributor Files (six) for Stafford-Clark cover the period 1953–1982. These files, labelled as ‘Personal Files’, are divided into two separate chronological series: four files for radio work (covering 1953–1982) and two for TV work (covering 1955–1970). The four radio files are abbreviated in text as: RCONT1; RCONT12/1; RCONT12/2; RCONT15. The two TV files are abbreviated in-text as: TVART1; TVART3.

Production Files contain programme and episode specific material relating to Stafford-Clark’s TV broadcasting on *Lifeline* and *Mind and Motive*. The file relating to *Brain and Behaviour* is missing. Thus: *Lifeline*: one general file (abbreviated T32/230), and ten episode files (abbreviated T32/875-84). The production files are incomplete since a number of episodes were not archived. *Mind and Motive*: one general file (abbreviated T50/68).

Microfilm of scripts for *Silver Lining*, *Lifeline*, *Brain and Behaviour*, and *Mind and Motive*: Runs are often incomplete, and many images are of poor quality and difficult to read. These are abbreviated as SLS and LLS ( *Brain and Behaviour* and *Mind and Motive* scripts have not been cited).

## Supplementary material

Supplementary File 1: Episode titles and first transmission for Stafford-Clark’s three BBC series, *Lifeline, Brain and Behaviour,* and *Mind and Motive*.

Supplementary File 2: Bibliography of material authored or co-authored by Stafford-Clark.
