# Supplementary material for: David Stafford-Clark (1916-1999): Seeing through a celebrity psychiatrist
Source: Wellcome Open Res. 2017 Apr 26;2:30. [Version 1] doi: 10.12688/wellcomeopenres.11411.1 (PMC5426535; doi:10.12688/wellcomeopenres.11411.1)
Supplement: Supplementary file 1 [file wellcomeopenres-2-12319-s0000.tgz › 8691f40f-2c46-4286-b22e-eebe1d53eb69.docx]

**Supplementary File 1: Episode titles and first transmission for Stafford-Clark’s three BBC series, *Lifeline, Brain and Behaviour,* and *Mind and Motive.***

A complete listing of all Stafford-Clark’s work for the BBC alone would be an enormous task, and is compounded by the corporation’s preference to bill him anonymously. The following listing provides episode titles and first transmission for Stafford-Clark’s three BBC TV series, *Lifeline*, *Brain and Behaviour*, and *Mind and Motive*. The information has been assembled from production files, and cross-checked again with information held in BBC Genome (<http://genome.ch.bbc.co.uk/>), an online searchable database of scanned listings information from *Radio Times*, 1923-2009.

*Lifeline* (all BBC Television; BBC 2 was not launched until April 1964)

Series 1

1. Offences Against Children Tuesday 15 October 1957

2. Is Religion Necessary? Tuesday 29 October 1957

3. The Story of Mr Prosser [on blindness] Tuesday 12 November 1957

4. The Problem of the Homosexual Tuesday 26 November 1957

5. Parent and Child Tuesday 10 December 1957

Series 2

6. Psychosomatic Illness Thursday 10 April 1958

7. Incurable Illness Thursday 24 April 1958

8. The Psychopath Thursday 22 May 1958

9. Brainwashing Thursday 5 June 1958

10. Young Offenders Thursday 19 June 1958

11. Plastic Surgery Thursday 3 July 1958

[Children in Hospital Thursday 17 July 1958, postponed by coverage of Middle East crisis to 14 August 1958]

12. Silent Order Thursday 31 July 1958

13. Young Children in Hospital Thursday 14 August 1958 [postponed from 17 July 1958]

14. The Battle against Leprosy Thursday 28 August 1958

15. Children of Divorced Parents Thursday 18 September 1958

Series 3

16. Corporal Punishment Thursday 1 January 1959

17. Christian Science Thursday 15 January 1959

18. Surgery of the Brain Thursday 29 January 1959

19. Mental Illness Thursday 12 February 1959

20. Termination of Pregnancy Thursday 26 February 1959

21. Battle for the Mind Thursday 12 March 1959

22. The Mystic Thursday 26 March 1959

23. Adolescence Thursday 9 April 1959

24. The Right to Die Thursday 16 April 1959

25. The Mongoloid Child Thursday 14 May 1959

26. Mars and Venus Speak to Earth Thursday 21 May 1959

27. Children and Violence Thursday 4 June 1959

28. False Pretenders Thursday 18 June 1959

Series 4

29. Fear Tuesday 13 October 1959

30. Hypnosis Tuesday 27 October 1959

31. Venereal Disease Tuesday 10 November 1959

32. The Subconscious Mind Tuesday 24 November 1959

33. Moment of Truth [near-death experience]Wednesday 6 January 1960

34. The Body Image Wednesday 20 January 1960

35. The Medium [part 1] Wednesday 3 February 1960

36. The Medium [part 2] Wednesday 17 February 1960

37. Extra-Sensory Perception Wednesday 2 March 1960

38. Hallucinations and Delusions Monday 25 April 1960

39. Schizophrenia Monday 9 May 1960

40. Obsessive Compulsions Monday 23 May 1960

41. Hysteria Monday 20 June 1960

*Lifeline* Special

42. AID [Artificial Insemination by Donor] Friday 29 July 1960

Series 5

[Hypnosis and the Unconscious Mind Friday 27 January 1961, postponed by DSC illness; replaced by ‘The Subconscious Mind’ above]

43. Hypnosis and the Unconscious Mind Friday 10 February 1961 [postponed from 27 Jan]

44. Reality and the Unconscious Friday 24 February 1961

45. Memory and the Unconscious Friday 10 March 1961

46. Free Will and the Unconscious Friday 24 March 1961

47. Judgement and the Unconscious Friday 7 April 1961

48. Medicine and the Unconscious Friday 21 April 1961

49. The Significance of the Unconscious Friday 5 May 1961

50. The Power of Faith Friday 19 May 1961

51. Epilepsy: The Sacred Disease Friday 2 June 1961

52. Alcoholism Friday 16 June 1961

53. Schizophrenia in Children [i.e. Autism] Friday 30 June 1961

*Lifeline* Special

54. Children under Stress 1: The Physical Symptoms Thursday 19 April 1962

55. Children under Stress 2: Problems at School Thursday 26 April 1962

56. Children under Stress 3: Trouble at Home Thursday 3 May 1962

*Brain and Behaviour* (BBC 2)

1. Us and Them Tuesday 11 August 1964

2. The Physical Framework Tuesday 18 August 1964

3. Instinct and Reaction Tuesday 25 August 1964

4. Reason over Reflex Tuesday 1 September 1964

5. The Emerging Pattern Tuesday 8 September 1964

6. The Adult under Stress Tuesday 15 September 1964

7. The Adult under Treatment Tuesday 22 September 1964

8. Them and Us Tuesday 29 September 1965

*Mind and Motive* (BBC 2)

1. Pain and Sadness Wednesday 11 January 1967

2. Cruelty and Anger Wednesday 18 January 1967

3. Love and Hate Wednesday 25 January 1967

4. Magic and Denial Wednesday 1 February 1967

5. Prejudice and Persecution Wednesday 8 February 1967

6. Courage and Desertion Wednesday 15 February 1967
