# Supplementary material for: David Stafford-Clark (1916-1999): Seeing through a celebrity psychiatrist
Source: Wellcome Open Res. 2017 Apr 26;2:30. [Version 1] doi: 10.12688/wellcomeopenres.11411.1 (PMC5426535; doi:10.12688/wellcomeopenres.11411.1)
Supplement: Supplementary file 2 [file wellcomeopenres-2-12319-s0001.tgz › a71b4ca5-c8b9-4093-a438-490f8e04a8ec.docx]

**Supplementary File 2: Bibliography of material authored or co-authored by Stafford-Clark.**

The following bibliography enumerates in date order published textual material authored or co-authored by Stafford-Clark. The list excludes non-Anglophone materials, editions other than first, and published correspondence to newspapers and periodicals. Given Stafford-Clark’s manifold writing activities, particularly for magazines and newspapers, the list is certainly incomplete. The items have been located from a variety of finding aids, including on-line databases, and Stafford-Clark’s Personal Papers (particularly the bibliography in PP/DSC/B/2/1, but also published material, press cuttings, page proofs, and drafts *passim*). Validation has been through inspection of electronic copies, paper copies, material in Stafford-Clark’s Personal Papers, and/or electronic bibliographic records.

Walter KH and Stafford-Clark D. (1938) Renal Function [part i]. *Guy’s Hospital Gazette* 52(1308): 351-354.

Walter KH and Stafford-Clark D. (1938) Renal Function [part ii]. *Guy’s Hospital Gazette* 52(1309): 372-374.

Stafford-Clark D. (1941) *Autumn Shadow and Other Poems,* Oxford: Shakespeare Head.

Stafford-Clark D. (1942-44) Combined Thomas Splint and Neil Robertson Stretcher in Rescue of Flying Casualties. *Medical Training Journal R.A.F.* i-iii.

Stafford-Clark D. (1943) Aspects of War Medicine in the RAF. *British Medical Journal* 1(4282): 139-140.

Stafford-Clark D. (1944) *Sound in the Sky and Other Poems,* Oxford: Basil Blackwell.

Stafford-Clark D. (1947) The Medico-Legal Problem of Homosexuality. *Medical Press and Circular* 218(10): 220-222.

Stafford-Clark D. (1947) The Teaching of First-Aid in the Air to Non-Medical Personnel in the Royal Air Force. In: Tidy HL and Kutschbach JMB (eds) *Inter-Allied Conferences on War Medicine 1942-1945.* London: Staples, 46-49.

Stafford-Clark D. (1948) Medical Emergencies in the Air. In: Birch CA (ed) *Emergencies in Medical Practice.* Livingstone: Edinburgh, 347-363.

Stafford-Clark D and Taylor FH. (1949) Clinical and Electro-encephalographic Studies of Prisoners Charged with Murder. *Journal of Neurology, Neurosurgery and Psychiatry* 12(4): 325-330.

Stafford-Clark D. (1949) Morale and Flying Experience: Results of a Wartime Study. *Journal of Mental Science* 95(398): 10-50.

Schwab RS and Stafford-Clark D. (1950) The Evaluation of Spontaneous Fasciculations in Conditions other than Progressive Muscular Atrophy. *Transactions of the American Neurological Association* 51: 169-174.

Stafford-Clark D. (1950) Painful Myostatic Dystonia: A Diagnostic Problem Treated by Psychotherapy and Unilateral Lobotomy. *American Practitioner and Digest of Treatment* 1(11): 1137-1143.

Stafford-Clark D. (1950) The Psychiatric Challenge in General Practice. *The Practitioner* 164(982): 355-360.

Stafford-Clark D. (1950) The Third Journey [Part i]. *Guy’s Hospital Gazette* 64(1611): 160-163.

Stafford-Clark D. (1950) The Third Journey [Part ii]. *Guy’s Hospital Gazette* 64(1612): 181-184.

Schwab RS, Stafford-Clark D and Prichard JS. (1951) The Clinical Significance of Fasciculations in Voluntary Muscle. *British Medical Journal* 2(4725): 209-212.

Stafford-Clark D, Pond D and Doust JL. (1951) The Psychopath in Prison: A Preliminary Report of a Co-operative Research. *The British Journal of Delinquency* 2(2): 117-129.

Stafford-Clark D. (1952) Crimes of Violence: The Report of a Conference on Crime Sponsored by the University of Colorado [Review]. *The British Journal of Delinquency* 2(3): 248-252.

Stafford-Clark D. (1952) Hypnotism and Hysteria. *Guy’s Hospital Gazette* 66(1667): 240-248.

Stafford-Clark D. (1952) Painful Myostatic Dystonia. In: Miles HHW, Cobb S and Shands HC (eds) *Case Histories in Psychosomatic Medicine.* New York: W.W. Norton, 217-235.

Stafford-Clark D. (1952) *Psychiatry To-day,* Harmondsworth, Middlesex: Penguin.

Stafford-Clark D. (1952) The Place of Psychiatry in Modern Medicine [Psychiatry Today - I]. *Guy’s Hospital Gazette* 66(1657): 24-30.

Stafford-Clark D. (1952) Psychiatry Today - II. *Guy’s Hospital Gazette* 66(1659): 73-78.

Stafford-Clark D. (1952) Psychiatry Today - III: Methods of Brief Psychotherapy. *Guy’s Hospital Gazette* 66(1662): 128-132.

Stafford-Clark D. (1952) Psychiatry Today - IV: The Wider Implications of Psychiatry. *Guy’s Hospital Gazette* 66(1664): 175-178.

Stafford-Clark D. (1953) The Diagnosis and Dangers of Depression. *Guy’s Hospital Gazette* 67(1697): 296-307.

Stafford-Clark D and Rolls EJ. (1954) Depersonalization Treated by Cannabis Indica and Psychotherapy. *Guy’s Hospital Reports* 103(4): 330-336.

Stafford-Clark D. (1954) The Diagnosis and Treatment of the Criminal Psychopath. *The Lancet* 263(6809): 459-460.

Stafford-Clark D. (1954) Epilepsy and Depression: Implications of Empirical Therapy. *Guy’s Hospital Reports* 103(4): 306-316.

Stafford-Clark D. (1954) The Etiology and Treatment of Impotence. *The Practitioner* 172(1030): 397-404.

Stafford-Clark D. (1954) What is Schizophrenia? (I). *Guy’s Hospital Gazette* 68(1728): 403-406.

Stafford-Clark D. (1954) What is Schizophrenia? (II). *Guy’s Hospital Gazette* 68(1729): 434-440.

Stafford-Clark D. (1955) The Concept of Psychopathic Personality - I. *Guy’s Hospital Gazette* 69(1758): 498-502.

Stafford-Clark D. (1955) The Concept of Psychopathic Personality - II. *Guy’s Hospital Gazette* 69(1759): 522-527.

Gibbens T, Pond D and Stafford-Clark D. (1955) A Follow-Up Study of Criminal Psychopaths. *The British Journal of Delinquency* 6(2): 126-136.

Stafford-Clark D. (1955) Principles and Practice in Psychiatric Treatment. *Case Conference* 1(10): 13-15.

Stafford-Clark D. (1955) Principles and Practice in Psychiatric Treatment II: Psycho-therapy. *Case Conference* 1(11): 10-12.

Stafford-Clark D. (1955) Principles and Practice in Psychiatric Treatment III: Psychoanalysis and Psychoanalytic Psychology. *Case Conference* 1(12): 17-20.

Stafford-Clark D. (1955) Principles and Practice in Psychiatric Treatment IV: Methods of Brief Psychotherapy. *Case Conference* 2(1): 12-14.

Stafford-Clark D. (1955) Principles and Practice in Psychiatric Treatment V: Physical Methods of Treatment. *Case Conference* 2(2): 19-21.

Stafford-Clark D. (1955) Principles and Practice in Psychiatric Treatment VI: Physical Methods of Treatment. *Case Conference* 2(3): 10-11.

Stafford-Clark D. (1955) Principles and Practice in Psychiatric Treatment. VII. Physical Methods of Treatment in Psychiatry. *Case Conference* 2(4): 10-12.

Stafford-Clark D. (1955) The Prognosis of Mental Illness Treated and Untreated. *Transactions of the Assurance Medical Society: Comprising the Report of the Proceedings for 1954-1955*: 1-18.

Stafford-Clark D. (1956) The Nature of the Problem. In: Mairet P (ed) *Christian Essays in Psychiatry.* New York: Philosophical Library, 13-28.

Stafford-Clark D. (1956) Psychiatry and the Law. In: Smith S (ed) *Taylor’s Principles and Practice of Medical Jurisprudence.* 11th ed. London: J & A Churchill, 545-609.

Stafford-Clark D. (1956) The Residents’ Play, 1956: ‘Kidney Punch’ or ‘The Changing Face of Guy’s’. *Guy’s Hospital Gazette* 70(1767): 109-127.

Stafford-Clark D. (1957) Battle for the Mind [Review]. *St Martin’s Review* (796): 205-208.

Garland H, Stafford-Clark D and Weatherall M. (1957) Discussion on the Toxic Effects of Drugs used in Neurological and Psychiatric Practice. *Proceedings of the Royal Society of Medicine* 50(8): 611-619.

Stafford-Clark D. (1957) Drug Action in Relation to Schizophrenia. *Modern Medicine of Great Britain* 2(7): 17-29.

Stafford-Clark D. (1957) Drug Action in Relation to Schizophrenia. In: Richter D (ed) *Schizophrenia: Somatic Aspects.* London: Pergamon, 163-178.

Stafford-Clark D. (1957) The Finchden Experiment [Review]. *Medical World* 86(1): 57-62.

Stafford-Clark D. (1957) Homosexuality. *Medico-Legal Journal* 25(2): 65-81.

Stafford-Clark D. (1957) Mental Health and Mental Disorder: A Sociological Approach [Review]. *Sociological Review* 5(1): 128-133.

Stafford-Clark D. (1957) Physics, Psychology and Medicine [Review]. *Bethlem & Maudsley Gazette.* 4-5.

Stafford-Clark D. (1957) The psychosomatic genesis of coronary artery disease [Review]. *Journal of Psychosomatic Research* 2(2): 152-154.

Stafford-Clark D. (1958) *British Red Cross Society Mental Health Manual,* London: British Red Cross Society.

Stafford-Clark D. (1958) Caring for Mentally Ill Patients. In: Houston J and Stockdale MG (eds) *Principles of Medicine and Medical Nursing.* London: English Universities Press, 173-177.

Stafford-Clark D. (1958) The Doctor’s Approach to Spiritual Healing. *The St Raphael Quarterly* 2(6): 533-544.

Stafford-Clark D. (1958) The Psychopath. In: Tredgold RF (ed) *Bridging the Gap: From fear to understanding in mental illness.* London: Christopher Johnson, 208-218.

Stafford-Clark D. [anon.] (1958) These Films Delve Among the Shadows. *Films and Filming.* 8, 34. (November).

Stafford-Clark D and Willis JH. (1959) Anorexia Nervosa. *The British Journal of Clinical Practice* 13(8): 533-540.

Stafford-Clark D. (1959) The Contribution of Psychiatry to Modern Medicine. *Journal of the Royal Society of Arts* 107(5040): 836-850.

Stafford-Clark D. (1959) A Follow-Up Study of Criminal Psychopaths. *Journal of Mental Science* 105(438): 108-115.

Stafford-Clark D. (1959) The Foundations of Research in Psychiatry. *British Medical Journal* 2(5161): 1199-1204.

Stafford-Clark D. (1959) *Mental Health and Illness,* London: Take Home.

Stafford-Clark D. (1959) The Psychiatric Challenge in General Practice. *The Almoner: A Journal of Medical Social Work* 12(9): 306-312.

Stafford-Clark D. [anon.] (1959) The Purpose of ‘Lifeline’. *The Listener.* 568-569. (8 October).

Stafford-Clark D. (1959) Thinking and Feeling. *The Listener.* 624-625. (15 October).

Stafford-Clark D. (1960) Foreword to Revised Edition. In: Hutton L *The Single Woman: Her Adjustment to Life and Love.* London: Barrie and Rockliff, v-vi.

Stafford-Clark D and Clarkson P. (1960) The Relationship of Appearance to Mental Illness. In: Wallace AB (ed) *The Transactions of the International Society of Plastic Surgeons: Second Congress.* Edinburgh: E & S Livingstone, 492-495.

Stafford-Clark D. (1960) Robert Waley Cohen Memorial Lecture 1960: The Psychology of Persecution & Prejudice. London: The Council of Christians and Jews.

Stafford-Clark D and Clarkson P. (1960) Role of the Plastic Surgeon and Psychiatrist in the Surgery of Appearance. *British Medical Journal* 2(5215): 1768-1771.

Stafford-Clark D. (1961) Hypnotism for medical and dental practitioners [Review]. *Journal of Psychosomatic Research* 5(2): 152-153.

Stafford-Clark D. (1961) The Integration of Services: a Summary of the York Clinic Experience. In: Linn L (ed) *Frontiers in General Hospital Psychiatry.* New York: International Universities Press, 3-26.

Stafford-Clark D. (1961) Ministerium Medici [Review]. *Journal of Psychosomatic Research* 5(3): 230.

Stafford-Clark D and Brook CPB. (1961) Psychiatric Treatment in General Wards. *The Lancet* 277(7187): 1159-1162.

Stafford-Clark D. (1961) The Psychology of Prejudice and Persecution. *Common Ground* 15(1): 4-10.

Stafford-Clark D, Hill D, Hinkle L, et al. (1961) Transatlantic Discussion: The Place of Psychiatry in Medicine To-day. *Proceedings of the Royal Society of Medicine* 54(12): 1079-1083.

Stafford-Clark D. [anon.] (1961) Women and their Doctors. *News of the World.* (12 March).

Stafford-Clark D. [anon.] (1961) When a Woman Falls in Love with her Doctor. *News of the World* (19 March)*.*

Stafford-Clark D. (1961-1962) Debate at Apothecaries Hall: ‘That Corporal Punishment is an Effective Deterrent against Crimes of Violence’. *The Transactions of the Hunterian Society* 20: 19-42.

Stafford-Clark D. (1962) Family Sickness and Anxiety. *The Royal Society of Health Journal* 82(4): 178-179.

Stafford-Clark D. (1962) Parent Education Through the Medium of Television. *Journal of the Medical Women’s Federation* 44(1): 16-19.

Stafford-Clark D. (1962) Psychiatry. In: Houston J, Joiner C and Trounce JR (eds) *A Short Textbook of Medicine.* London: English Universities Press, 5-59.

Stafford-Clark D. (1962) Suffering and Character. In: Rose ME (ed) *The Problem of Suffering: A collection of essays based on a series of broadcast talks for sixth forms, provided by the BBC under the general title The Christian Religion and its Philosophy.* London: British Broadcasting Corporation, 105-112.

Stafford-Clark D. (1962) Techniques of Hypnotherapy [Review]. *Journal of Psychosomatic Research* 6(4): 302.

Stafford-Clark D. (1962) [untitled, on topic ‘Diagnosis?’]. *Violence and the Mental Health Services: proceedings of a conference held by the National Association for Mental Health at Church House, Westminister, London, on 8th and 9th March, 1962.* London: National Association for Mental Health, 36-43.

Butterfield J, Mallott BL and Stafford-Clark D. (1963) Disorientation: An Approach to the Psychophysiology of Confusion, with Particular Reference to Psychiatric Emergencies in General Hospitals. *Proceedings of the The Third World Congress of Psychiatry.* No place: University of Toronto / McGill University, 506-511.

Stafford-Clark D. (1963) History Excellent, Diagnosis Misleading, Prognosis Uncertain [Review]. *New Scientist.* (14 March).

Stafford-Clark D. (1963) The Manipulation of Human Behaviour [Review]. *Journal of Psychosomatic Research* 7(3): 249-250.

Stafford-Clark D. (1963) Not Interred with their Bones [Review]. *New Scientist.* (28 November)

Stafford-Clark D. (1963) The Psychiatric Patient as an Insurance Risk. *Transactions of the Assurance Medical Society: comprising the report of the proceedings for 1962 to 1963*: 59-75.

Stafford-Clark D and Brook CPB. (1963) Psychiatric Treatment in General Wards. In: Freeman H and Farndale J (eds) *Trends in the Mental Health Service: A Symposium of Original and Reprinted Papers.* London: Pergamon, 111-118.

Stafford-Clark D. (1963) ‘Throw Physic to the Dogs ...’ [Review]. *New Scientist.* (11 April).

Stafford-Clark D. (1964) Closed Circuit Television in Psychiatric Teaching and Research. *The University Film Journal* (25-26): 20-21.

Stafford-Clark D. (1964) Essentials of the Clinical Approach. In: Rosen I (ed) *The Pathology and Treatment of Sexual Deviation: a methodological approach.* London: Oxford University Press, 57-86.

Stafford-Clark D. (1964) Law, Liberty and Psychiatry [Review]. *Yale Law Journal* 74(2): 392-399.

Stafford-Clark D. (1964) Plea for Medicine [Review]. *The Guardian.* (6 March).

Stafford-Clark D. (1964) *Psychiatry for Students ... With a chapter on child psychiatry by Gerard Vaughan ... and an appendix on clinical psychology by Jessie Williams,* London: George Allen & Unwin.

Stafford-Clark D. (1964) Psychological Reprints [Review]. *The British Journal of Psychiatry* 110(469): 872.

Stafford-Clark D. (1964) The Role of the Consultant In Tranquilizer Therapy. *Journal of Neuropsychiatry* 5(7): 448-455.

Stafford-Clark D. (1964-1965) Parapsychology and Pharmacology. *Parapsychology* 6(3): 72-79.

Brough DI, Yorkston N and Stafford-Clark D. (1965) A case of wasp phobia treated by systematic desensitization under light hypnosis. *Guy’s Hospital Reports* 114(3): 319-324.

Loucas KP and Stafford-Clark D. (1965) Electronarcosis at Guy’s. *Guy’s Hospital Reports* 114(3): 223-237.

Stafford-Clark D. (1965) Problems of Sex in General Practice [contribution to symposium]. *The Journal of the College of General Practitioners* 9(supplement 1): 1-4.

Stafford-Clark D. (1965) Psychiatric Emergencies. In: Gardiner-Hill H (ed) *Compendium of Emergencies.* 2nd ed. London: Butterworths, 147-160.

Stafford-Clark D. (1965) Psychiatry at Guy’s Hospital (II). *Guy’s Hospital Reports* 114(3): 151-173.

Stafford-Clark D. (1965) Psychosomatic Implications of Obsessive-Compulsive Disorders and Their Resemblance to Certain Types of Central Pain. *Guy’s Hospital Reports* 114(3): 209-222.

Stafford-Clark D. (1965) Psychosomatic Implications of Obsessive-Compulsive Disorders and Their Resemblance to Certain Types of Central Pain. *Journal of Psychosomatic Research* 9(1): 93-101.

Stafford-Clark D. (1965) [untitled, on ‘Eskimo Nell’ lecture]. *The Twentieth Century* 174: 17-22.

Stafford-Clark D. (1965) *What Freud Really Said,* London: Macdonald.

Stafford-Clark D. [anon.] (1966) Frigidity. *Nova.* 56-58. (January).

Stafford-Clark D. (1966) A Consumer’s Guide to the Do-it-yourself Sex Books. *Nova.* 22-27. (July).

Stafford-Clark D. (1966) Modern Discoveries in Medical Psychology [Review]. *The British Journal of Psychiatry* 112(487): 648.

Stafford-Clark D. (1966) The Nature of Aggression. *International Nursing Review* 13(3): 23-24.

Stafford-Clark D. (1966) The Nature of Aggression. *Mental Health* 25(1): 3-4.

Stafford-Clark D. (1966) Odious Letters. *Christian Action.* 9-15. (Autumn).

Stafford-Clark D. (1966) Psychiatry and Guy’s Hospital. *Guy’s Hospital Gazette* 80(2036): 388-395.

Stafford-Clark D. (1967) City Psychiatric [Review]. *The British Journal of Psychiatry* 113(502): 1052-1054.

Stafford-Clark D. (1967) Drug Dependence 1: The Significance of Human Vulnerability. *The Times.* (11 April).

Stafford-Clark D. (1967) Drug Dependence 2: Disposing of the myths and half-truths. *The Times.* (12 April).

Stafford-Clark D. (1967) Foreword. In Freeman, G *The Undergrowth of Literature.* London: Thomas Nelson, xi-xxvi.

Stafford-Clark D. (1967) LSD’s place is the hospital not the beat club. *The Times.* (17 February).

Stafford-Clark D. [anon.] (1967) The Man at the Maudsley. *The Time Literary Supplement.* 1213. (14 December).

Stafford-Clark D and Comfort A. (1967) Mind and Body: Dr David Stafford-Clark and Dr Alex Comfort talk with Peter Snow. In: *Dialogue with Doubt: ‘Last Programmes’ from Rediffusion Television.* London: SCM, 121-146.

Stafford-Clark D. (1967) Prejudice in the Community. London: National Committee for Commonwealth Immigrants.

Stafford-Clark D. (1967) Second Opinion: Maurice Girodias. *Sunday Times Magazine.* (12 February).

Stafford-Clark D. (1968) The Credibility Gap: Religious experience - or Illusion? Cambridge: Great Saint’s Mary Church.

Stafford-Clark D. (1968) The Dark Mirror, etc. [Reviews]. *The British Journal of Psychiatry* 114(516): 1465-1467.

Stafford-Clark D. (1968) How tycoons and tigers respond to stress and tension. *The Times.* (25 January).

Stafford-Clark D. [anon.] (1968) Possessiveness: the Vice that Masquerades as Virtue. *Good Housekeeping.* 38-39, 93. (January)

Stafford-Clark D. (1968) The Psychology of Prejudice. In: Hill CS and Mathews D (eds) *Race: A Christian Symposium.* London: Victor Gollancz, 69-88.

Stafford-Clark D. (1969) The Arithmetic of a Brave New World. *The Times.* (3 April).

Stafford-Clark D. (1969) Brain Surgery’s Fundamental Limitations. *The Times.* (21 May).

Stafford-Clark D. (1969) A Letter to the Editor. In: Blom-Cooper L (ed) *The Hanging Question: Essays on the Death Penalty.* London: Gerald Duckworth, 127-132.

Stafford-Clark D. (1970) Current Theories on Conscience and Consciousness. *Proceedings of the Royal Institution of Great Britain* 43(200): 112-136.

Stafford-Clark D. (1970) *Five Questions in Search of an Answer: Religion and Life: Some Inescapable Contradictions,* London: Thomas Nelson.

Stafford-Clark D. (1970) The Human Predicament [Review]. *The British Journal of Psychiatry* 116(530): 99-102.

Stafford-Clark D. (1970) Real Mesmeriser [Review]. *Sunday Telegraph.* (5 July).

Stafford-Clark D. (1970) Recent Advances in Psychiatry. In: Horrobin D and Gunn A (eds) *The International Handbook of Medical Science: A Concise Guide to Current Practice and Recent Advances.* Aylesbury: MTP, 51-75.

Stafford-Clark D. (1970) Supportive Psychotherapy. In: Price JH (ed) *Modern Trends in Psychological Medicine.* London: Butterworths, 277-295.

Stafford-Clark D. (1972) A Guy’s/Maudsley Special Out-Patient Clinic: Evolution from January 1956 to September 1971. *Guy’s Hospital Reports* 121(2-3): 179-192.

Stafford-Clark D. (1972) The Reality of Violence. *Illustrated London News.* London, England, 27-28. (30 December).

Stafford-Clark D. (1974) Decent is as Decent Looks. *Illustrated London News.* London, England, 27. (26 January).

Stafford-Clark D. [as ‘DS-C’] (1974) Psychiatric Treatment, Concepts of. *Encyclopaedia Britannica.* 15th ed. Chicago: Encyclopaedia Britannica Inc., 141-148.

Stafford-Clark D. (1974) Seeing and Believing. *Illustrated London News.* London, England, 47. (27 April).

Stafford-Clark D. (1976) A Mind for All Reasons [Review]. *Psychology Today.* 33-35. (March).

Stafford-Clark D. (1979) *Soldier Without a Rifle,* London: Collins.

Stafford-Clark D. (1983) The Advance of Nuclear Medicine. *Illustrated London News.* London, England, 25. (31 December).

Stafford-Clark D. (1984) Personality of a Hospital: The Guy’s Story from 1948 to 1982. *Guy’s Hospital Gazette* 98(2339): 12-27.

Stafford-Clark D. (1985) What Makes a Champion? *Illustrated London News.* London, England, 58-60. (29 June).

Stafford-Clark D. (1987) An Hour of Breath. *Bulletin of the Royal College of Psychiatrists* 11(7): 218-223.

Stafford-Clark D. (1995) A Strange Deceit. *Psychiatric Bulletin* 19(8): 504-505.
