# Supplementary material for: David Stafford-Clark (1916-1999): Seeing through a celebrity psychiatrist
Source: Wellcome Open Res. 2017 Apr 26;2:30. [Version 1] doi: 10.12688/wellcomeopenres.11411.1 (PMC5426535; doi:10.12688/wellcomeopenres.11411.1)
Supplement: Peer review history

# Peer review: Referee response for version 1

**DOI:** 10.21956/wellcomeopenres.12319.r22580

**Published:** 2017-05-09

**Authors:** Anne Karpf

**Affiliations:** 1 Sir John Cass School of Art, Architecture and Design, London Metropolitan University, London, UK

This is a well-researched, illuminating article on the media rise and fall of a celebrity psychiatrist. The author uses this case history to explore 'the economy of celebrity' in the mid-20th century, looking at the ways in which the psychiatrist David Stafford-Clark exploited the growing cultural authority of psychiatry in the 1950s and early 1960s at a time when the expansion of broadcasting created opportunities for white, male, middle-aged public intellectuals endowed with cultural gravitas from their expertise in another sphere. The article analyses Stafford-Clark's role in promoting psychiatry as a legitimating force even on topics on which it - and he - had little or no expertise. He thus became a vector for medicalisation - paradoxically, even on subjects such as homosexuality where he argued against a medicalised view.

 The article shows, with great skill, how Stafford-Clark's self-aggrandising project was eventually the cause of his undoing, positioning him no longer as an expert but now as a quasi- or ersatz- broadcaster, albeit one who did not conform to the norms of the medium in his propensity to moralise or talk at the audience.

 The article makes a valuable contribution, therefore, to the history of the medical profession in broadcasting and the difficulties they faced in reconciling conflicting imperatives. If I may I'd like to suggest that Stafford-Clark's career could be contrasted with that of the British psychoanalyst Donald Winnicott. Although Winnicott broadcast over the radio and not television, through the fifty radio broadcasts that he made between 1943 and 1962 he also attained fame beyond the consulting-room, yet he scrupulously avoided Stafford-Clark's moralising tone. He also refrained from intervening over the airwaves in controversial debates, despite being outspoken on subjects such as leucotomy in the medical press, and thus protected his professional reputation ^1^.

 Miller is bold in his critique of Stafford-Clark's increasingly narcissistic relationship with broadcasting and provides ample evidence to substantiate it. There are many future lines of inquiry that could stem from this original and well-written piece of research which, I hope, will form part of a larger study.

I have read this submission. I believe that I have an appropriate level of expertise to confirm that it is of an acceptable scientific standard.

## References

[1] KarpfA: Constructing and Addressing the 'Ordinary Devoted Mother'. *History Workshop Journal*.2014;78(1) : 10.1093/hwj/dbt037 82-106 10.1093/hwj/dbt037

# Peer review: Referee response for version 1

**DOI:** 10.21956/wellcomeopenres.12319.r22476

**Published:** 2017-05-05

**Authors:** Mathew Thomson

**Affiliations:** 1 Department of History, University of Warwick, Coventry, UK

It was a great pleasure to read this engaging, informative, and thought-provoking essay. David Stafford-Clark is brought to life as an intriguing character through impressive original research and provocative and intelligent analysis. The essay draws on Stafford-Clark’s voluminous scattered publications, but also his revealing personal papers and the records on his radio and television contributions held in the BBC written archives. Listings of Stafford-Clark’s publications and broadcast output are both included with the essay and will be of value to future research.

 Stafford-Clark has attracted little previous attention, but as the essay clearly demonstrates he was in fact a significant figure in post-war British public life. In explaining how this came about, and in uncovering what Stafford-Clark did with this position, Miller helps us to understand how psychiatry developed a public face in post-war Britain and explains convincingly the difficulties that arose in combining the roles of expert psychiatrist and public intellectual (which would resurface in the better known public life of R.D. Laing in the years that followed). We are led towards this understanding through analysis of Stafford-Clark’s publishing career (including his efforts to develop the reputation as a poet) and even more crucially through his rise (and fall) as ‘the BBC psychiatrist’. This is a richly detailed and well told biographical story based on excellent research. The essay is also an important contribution in bringing psychiatry into the broader story of the intellectual life of the post-war nation, and it engages with an impressive range of secondary literature in opening up this subject. It is work therefore that deserves the attention not just of historians of medicine, psychiatry and the development of a psychological society, but also of a broader body of historians interested in the impact of the social sciences, the media, secularisation, and the emergence of a permissive society in post-war Britain. Broader still, experts in none of these will find this a fascinating story and an enjoyable read.

I have read this submission. I believe that I have an appropriate level of expertise to confirm that it is of an acceptable scientific standard.
